# Prediction and prognosis of adverse maternal and foetal/neonatal outcomes in pulmonary hypertension: an observational study and nomogram construction

**DOI:** 10.1186/s12931-022-02235-y

**Published:** 2022-11-15

**Authors:** Yuqin Chen, Dansha Zhou, Mingmei Xiong, Xin Xi, Wenni Zhang, Ruifeng Zhang, Lishi Chen, Qian Jiang, Ning Lai, Xiang Li, Jieer Luo, Xuanyi Li, Weici Feng, Chuhui Gao, Jiyuan Chen, Xin Fu, Wei Hong, Mei Jiang, Kai Yang, Wenju Lu, Yiping Luo, Jun Zhang, Zhe Cheng, Chunli Liu, Jian Wang

**Affiliations:** 1 State Key Laboratory of Respiratory Diseases, Guangdong Key Laboratory of Vascular Diseases, National Clinical Research Centre for Respiratory Diseases, Guangzhou Institute of Respiratory Health, The First Affiliated Hospital of Guangzhou Medical University, 151 Yanjiang Road, Guangzhou, 510120 Guangdong People’s Republic of China; 2 The Third Affiliated Hospital of Guangzhou Medical University, Guangzhou, 510140 Guangdong People’s Republic of China; 3 Sleep Centre and Department of Respiratory Medicine, Beijing Anzhen Hospital of Capital Medical University, Beijing, 100029 People’s Republic of China; 4 Guangdong Women and Children’s Hospital, 521 Xingnan Avenue, Panyu District, Guangzhou, 511442 Guangdong People’s Republic of China; 5 Department of Respiratory Medicine, Zhongda Hospital of Southeast University, Nanjing, 210009 People’s Republic of China; 6 GMU-GIBH Joint School of Life Sciences, Guangzhou Medical University, Guangzhou, Guangdong People’s Republic of China; 7 Pulmonary and Critical Care Medicine, The First Affiliated Hospital of Zhengzhou University, Zhengzhou, 450052 Henan People’s Republic of China; 8 Section of Physiology, Division of Pulmonary, Critical Care and Sleep Medicine, University of California, San Diego, La Jolla, CA USA

**Keywords:** Pregnancy, Pulmonary hypertension, Prediction model, Prognostic model, Overall survival, Machine-based model, Nomogram

## Abstract

**Background:**

Pregnant women with pulmonary hypertension (PH) have higher mortality rates and poor foetal/neonatal outcomes. Tools to assess these risk factors are not well established.

**Methods:**

Predictive and prognostic nomograms were constructed using data from a “Development” cohort of 420 pregnant patients with PH, recorded between January 2009 and December 2018. Logistic regression analysis established models to predict the probability of adverse maternal and foetal/neonatal events and overall survival by Cox analysis. An independent “Validation” cohort comprised data of 273 consecutive patients assessed from January 2019 until May 2022. Nomogram performance was evaluated internally and implemented with online software to increase the ease of use.

**Results:**

Type I respiratory failure, New York Heart Association functional class, N-terminal pro-brain natriuretic peptide $\ge$ 1400 ng/L, arrhythmia, and eclampsia with pre-existing hypertension were independent risk factors for maternal mortality or heart failure. Type I respiratory failure, arrhythmia, general anaesthesia for caesarean section, New York Heart Association functional class, and N-terminal pro-brain natriuretic peptide $\ge$ 1400 ng/L were independent predictors of pulmonary hypertension survival during pregnancy. For foetal/neonatal adverse clinical events, type I respiratory failure, arrhythmia, general anaesthesia for caesarean section, parity, platelet count, fibrinogen, and left ventricular systolic diameter were important predictors. Nomogram application for the Development and Validation cohorts showed good discrimination and calibration; decision curve analysis demonstrated their clinical utility.

**Conclusions:**

The nomogram and its online software can be used to analyse individual mortality, heart failure risk, overall survival prediction, and adverse foetal/neonatal clinical events, which may be useful to facilitate early intervention and better survival rates.

**Supplementary Information:**

The online version contains supplementary material available at 10.1186/s12931-022-02235-y.

## Background

Pulmonary hypertension (PH) is a chronic, progressive cardiopulmonary disease with a significant risk of maternal and foetal/neonatal complications [1, 2]. According to the 2018 European Society of Cardiology (ESC) guidelines, the mortality rate of pregnant patients with PH is approximately 16–30% [3], with most deaths occurring in the first week postpartum [2, 4]. Foetal/neonatal outcomes are also reported to be poor, with mortality rates ranging from 7 to 13% [5]. Therefore, the guidelines recommend against pregnancy, favour contraception, and encourage early pregnancy termination in these patients [3]. Nevertheless, the pregnancy rate among women with PH is increasing [6], with some patients even declining termination. Moreover, 30% of women are diagnosed with PH during pregnancy, presenting a challenge for mothers and caregivers [7, 8].

Advances have been made in pharmacological and other treatments for PH, improving the overall quality of life and prognosis [9] and lowering mortality rate (3%) [2]. However, because the maternal mortality rate remains high, pregnancy remains contraindicated in women with PH [3]. A large-scale, multi-centre clinical study is needed to reconsider this general proscription. In the meantime, pregnant patients with PH must be informed regarding their options and pregnancy-associated risks.

To date, no objective, large-scale, multi-centre, machine learning-based clinical prediction models have been developed to evaluate the adverse maternal and foetal/neonatal outcomes in pregnant patients with PH. Given the risks, practical and reliable tools for early assessment of adverse foetal/neonatal events, maternal mortality or heart failure (HF), and overall survival (OS) are needed. Nomograms are based on core diagnostic indicators and are useful for comprehensive patient evaluation and early disease diagnosis. They have been effectively applied for various conditions, including soft-tissue sarcomas [10], small-cell lung cancer [11], coronavirus disease [12], and aortic dissection [13].

In this study, we aimed to develop and validate a machine-based model to predict maternal mortality or HF and adverse foetal/neonatal clinical events in pregnant patients with PH. The goal is to identify high-risk patients and make rapid, accurate clinical decisions. Additionally, we constructed and verified a prognostic model to guide treatment, advancing the capability for early intervention.

## Methods

### Patients

We reviewed obstetric records from six Chinese hospitals from 2009 to 2022. PH was defined according to the clinical diagnostic criteria, with pulmonary artery systolic pressure > 35 mmHg [14, 15] confirmed by echocardiography. Maternal mortality was defined as death during pregnancy or within 7 days postpartum. HF was defined according to the ESC and American Heart Association (AHA) guidelines [16, 17], as a complex clinical syndrome caused by any structural or functional damage resulting from ventricular filling or blood excretion. Patients with elevated right ventricular systolic pressure from outflow obstruction or pulmonary stenosis were excluded. The Development cohort included 420 patients who met these criteria between January 2009 and December 2018. An independent Validation cohort comprising 273 consecutive patients from January 2019 to May 2022 was created using the same criteria. Both cohorts were observed over time, with 117 (16.8%) patients lost to follow-up. The remaining 355 patients from the Development cohort were used as the Follow-up set for the OS nomogram construction, and the remaining 221 patients from the Validation cohort became the external validation set for the prognostic nomogram. Pregnancy was continued in 304 patients from the Development cohort and in 194 patients from the Validation cohort. The corresponding foetal/neonatal records were included in the Delivery and Validation groups, respectively. These groups were used to establish (and validate) another nomogram to predict adverse foetal/neonatal events. This study protocol was approved by the Medical Ethics Committee of the Guangdong Women and Children Hospital (reference number: 202101357). The requirement for informed consent was waived due to the retrospective nature of the study.

### Patient characteristics and outcome measures

The primary outcome was maternal mortality or HF. OS was calculated from diagnosis to all-cause death or final follow-up (May 2022). Foetal/neonatal death and adverse clinical events were a composite of foetal death (in utero), neonatal death (within 30 days of birth), and small for gestational age (SGA) (foetal/neonatal weight of small for gestational age < 10%) [18].

### Statistical analysis

Stepwise regression based on the minimum value of the Akaike information criterion was used to select variables for nomogram inclusion [19]. Discriminatory ability was assessed using the bootstrap concordance index (C-index) and area under the curve (AUC) of the receiver operating characteristic (ROC) curve [20]. The Hosmer–Lemeshow test versus the calibration curve was used to evaluate the ability to calibrate [21]. A C-index or AUC value > 0.7 indicated that the nomogram had good discriminatory ability [22]. The calibration plot showed the predicted and actual probabilities for each patient in the nomogram model, with a line close to the ideal 45° indicating good correlation [23, 24]. Decision curve analysis (DCA) was performed to evaluate the clinical utility of the nomogram [25, 26]. Detailed and expanded methods are found in the Online Supplement.

## Results

### Patient and disease characteristics

The maternal mortality rates in the Development and the Validation cohorts were 10.2% (43/420) and 4.4% (12/273), respectively, while the probabilities of HF were 16.2% (68/420) and 12.8% (35/273), respectively. The foetal/neonatal mortality rates in the Delivery and Validation groups were 6.6% (20/304) and 3.5% (8/228), respectively; the rates of SGA were 41.4% (126/304) and 32.9% (75/228), respectively. The demographic and clinical characteristics and univariate logistics analyses of the Development and Validation cohorts are summarised in Table 1, and those of the Follow-up and Validation sets are shown in Table 2 and Additional file 1: Table S1, respectively. Maternal and foetus/neonate characteristics in the Delivery and Validation groups are provided in Additional file 1: Table S2.

**Table 1:** Demographic and clinical characteristics of patients in the Development and External validation cohorts

Variable	Development cohort				External validation cohort		
	Non-death or HF (n = 309)	Death or HF (n = 111)		*P* value	Non-death or HF (n = 226)	Death or HF (n = 47)	*P* value
Age, median (IQR), year	29.0 (25.0–33.0)	28.0 (25.0–32.0)		0.211	30.0 (28.0–34.3)	30.0 (27.0–32.0)	0.118
Mild preeclampsia, No. (%)							
No	305 (98.7)	108 (97.3)		0.331	213 (94.2)	45 (95.7)	0.683
Yes	4 (1.3)	3 (2.7)			13 (5.8)	2 (4.3)	
Severe preeclampsia, No. (%)							
No	268 (86.7)	84 (75.7)		0.007	184 (81.4)	34 (72.3)	0.161
Yes	41 (13.3)	27 (24.3)			42 (18.6)	13 (27.7)	
Eclampsia with pregnancy/delivery, No. (%)							
No	281 (90.9)	88 (79.3)		0.002	198 (87.6)	44 (93.6)	0.247
Yes	28 (9.1)	23 (20.7)			28 (12.4)	3 (6.4)	
Eclampsia with pre-existing HTN, No. (%)							
No	298 (96.4)	98 (88.3)		0.003	215 (95.1)	43 (91.5)	0.325
Yes	11 (3.6)	13 (11.7)			11 (4.9)	4 (8.5)	
Postpartum haemorrhage, No. (%)							
No	296 (95.8)	105 (94.6)		0.270	206 (91.2)	46 (97.9)	0.149
Yes	13 (4.2)	6 (5.4)			20 (8.8)	1 (2.1)	
Multiple pregnancies, No. (%)							
No	294 (95.1)	104 (93.7)		0.557	197 (87.2)	42 (89.4)	0.679
Yes	15 (4.9)	7 (6.3)			29 (12.8)	5 (10.6)	
Premature rupture of membranes, No. (%)							
No	293 (94.8)	105 (94.6)		0.927	208 (92.0)	44 (93.6)	0.712
Yes	16 (5.2)	6 (5.4)			18 (8.0)	3 (6.4)	
Type I respiratory failure, No. (%)							
No	306 (99.0)	85 (76.6)		< 0.001	220 (97.3)	30 (63.8)	< 0.001
Yes	3 (10.0)	26 (23.4)			6 (2.7)	17 (36.2)	
Type II respiratory failure, No. (%)							
No	309 (100.0)	108 (97.3)		0.999	225 (99.6)	46 (97.9)	0.265
Yes	0 (0.0)	3 (2.7)			1 (0.4)	1 (2.1)	
Arrhythmia, No. (%)							
No	208 (67.3)	52 (46.8)		< 0.001	151 (66.8)	23 (48.9)	0.022
Yes	101 (32.7)	59 (53.2)			75 (33.2)	24 (51.1)	
Patent ductus arteriosus, No. (%)							
No	297 (96.1)	104 (93.7)		0.297	217 (96.0)	44 (93.6)	0.469
Yes	12 (3.9)	7 (6.3)			9 (4.0)	3 (6.4)	
Ventricular septal defect, No. (%)							
No	258 (83.5)	88 (79.3)		0.318	204 (90.3)	42 (89.4)	0.850
Yes	51 (16.5)	23 (20.7)			22 (9.7)	5 (10.6)	
Atrial septal defect, No. (%)							
No	213 (68.9)	99 (89.2)		< 0.001	181 (80.1)	44 (93.6)	0.037
Yes	96 (31.1)	12 (10.8)			45 (19.9)	3 (6.4)	
Pulmonary embolism, No. (%)							
No	305 (98.7)	107 (96.4)		0.143	224 (99.1)	47 (100.0)	0.999
Yes	4 (1.3)	4 (3.6)			2 (0.9)	0 (0.0)	
Endocarditis, No. (%)							
No	307 (99.4)	110 (99.1)		0.786	226 (100.0)	46 (97.9)	1.000
Yes	2 (0.6)	1 (0.9)			0 (0.0)	1 (2.1)	
Myocardiopathy, No. (%)							
No	301 (97.4)	101 (91.0)		0.007	226 (100.0)	44 (93.6)	0.999
Yes	8 (2.6)	10 (9.0)			0 (0.0)	3 (6.4)	
Rheumatic heart disease, No. (%)							
No	280 (90.6)	97 (87.4)		0.338	217 (96.0)	45 (95.7)	0.938
Yes	29 (9.4)	14 (12.6)			9 (4.0)	2 (4.3)	
Congenital heart disease, No. (%)							
No	127 (41.1)	62 (55.9)		< 0.001	140 (61.9)	35 (74.5)	0.107
Yes	182 (58.9)	49 (44.1)			86 (38.1)	12 (25.5)	
Eisenmenger syndrome, No. (%)							
No	294 (95.1)	90 (81.1)		< 0.001	215 (95.1)	44 (93.6)	0.669
Yes	15 (4.9)	21 (18.9)			11 (4.9)	3 (6.4)	
Gestational diabetes mellitus, No. (%)							
No	281 (90.9)	88 (79.3)		0.002	193 (85.4)	44 (93.6)	0.142
Yes	28 (9.1)	23 (20.7)			33 (14.6)	3 (6.4)	
Infection, No. (%)							
No	281 (90.9)	76 (68.5)		< 0.001	181 (80.1)	24 (51.1)	< 0.001
Yes	28 (9.1)	35 (31.5)			45 (19.9)	23 (48.9)	
Systemic lupus erythematosus, No. (%)							
No	301 (97.4)	106 (95.5)		0.324	216 (95.6)	41 (87.2)	0.034
Yes	8 (2.6)	5 (4.5)			10 (4.4)	6 (12.8)	
Liver insufficiency, No. (%)							
No	303 (98.1)	109 (98.2)		0.926	217 (96.0)	44 (93.6)	0.469
Yes	6 (1.9)	2 (1.8)			9 (4.0)	3 (6.4)	
Left to right shunt, No. (%)							
No	293 (94.8)	107 (96.4)		0.506	211 (93.4)	44 (93.6)	0.949
Yes	16 (5.2)	4 (3.6)			15 (6.6)	3 (6.4)	
Right-to-left shunt, No. (%)							
No	301 (97.4)	107 (96.4)		0.584	220 (97.3)	46 (97.9)	0.836
Yes	8 (2.6)	4 (3.6)			6 (2.7)	1 (2.1)	
Premature delivery, No. (%)							
No	130 (42.1)	37 (33.3)		0.803	93 (59.3)	17 (59.6)	0.193
Yes	112 (36.2)	49 (44.1)			100 (11.9)	18 (23.4)	
PH classification, No. (%)							
Group 1	265 (85.8)	84 (75.7)		0.132	199 (88.1)	40 (85.1)	0.760
Group 2	37 (12.0)	20 (18.0)			17 (7.5)	4 (8.5)	
Group 3	1 (0.3)	1 (0.9)			3 (1.3)	0 (0.0)	
Group 4	1 (0.3)	2 (1.8)			0 (0.0)	0 (0.0)	
Group 5	5 (1.6)	4 (3.6)			7 (3.1)	3 (6.4)	
Pregnancy outcome, No. (%)							
Termination of pregnancy	67 (21.7)	25 (22.5)		< 0.001	33 (14.6)	12 (25.5)	0.017
Vaginal delivery	20 (6.5)	8 (7.2)			15 (6.6)	1 (2.1)	
Spinal and/or epidural for C-section	204 (66.0)	46 (41.4)			172 (76.1)	29 (61.7)	
General anaesthesia for C-section	18 (5.8)	32 (28.8)			6 (2.7)	5 (10.6)	
Cardiac surgery, No. (%)							
No	292 (94.5)	109 (98.2)		0.127	204 (90.3)	46 (97.9)	0.171
Single lung transplantation	0 (0.0)	0 (0.0)			5 (2.2)	0 (0.0)	
Repair of heart defect	17 (5.5)	2 (1.8)			17 (7.5)	1 (2.1)	
NYHA functional class, No. (%)							
I / II	215 (69.6)	23 (20.7)		< 0.001	184 (81.4)	13 (27.7)	< 0.001
III	73 (23.6)	41 (36.9)			31 (13.7)	11 (23.4)	
IV	21 (6.8)	47 (42.3)			11 (4.9)	23 (48.9)	
NT-proBNP, No. (%), ng/L							
< 1400	277 (89.6)	43 (38.7)		< 0.001	199 (88.1)	17 (36.2)	< 0.001
$\ge$1400	32 (10.4)	68 (61.3)			27 (11.9)	30 (63.8)	
Gestation times, median (IQR), times	2.0 (1.0–3.0)	2.0 (1.0–3.0)		0.006	2.0 (1.0–3.0)	2.0 (1.0–3.0)	0.811
Parity, median (IQR), times	1.0 (1.0–2.0)	1.0 (1.0–2.0)		0.690	1.0 (1.0–2.0)	2.0 (1.0–2.0)	0.507
Troponin, median (IQR), ng/mL	0.01 (0.00–0.01)	0.03 (0.00–0.20)		0.185	0.01 (0.00–0.10)	0.02 (0.01–5.70)	0.164
Prothrombin time, median (IQR), s	10.1 (9.5–10.8)	10.4 (9.6–11.7)		< 0.001	10.4 (9.7–11.1)	10.7 (9.8–11.7)	0.009
APTT, median (IQR), s	28.9 (26.5–31.6)	30.2 (27.6–34.1)		< 0.001	27.6 (25.9–30.4)	29.8 (26.7–32.9)	0.009
Thrombin time, median (IQR), s	14.1 (12.8–16.5)	13.9 (12.9–16.3)		0.038	14.4 (13.2–16.0)	14.3 (13.1–16.1)	0.089
Fibrinogen, median (IQR), g/L	3.7 (3.1–4.3)	3.4 (2.8–4.0)		0.028	3.7 (3.3–4.3)	3.8 (3.0–4.7)	0.028
RBC, median (IQR), * 10^12/L	3.9 (3.5–4.3)	3.9 (3.3–4.3)		0.218	3.8 (3.5–4.2)	3.6 (3.2–4.3)	0.617
Haemoglobin, median (IQR), g/L	113.0 (102.0–124.0)	106.0 (93.0–123.0)		0.007	111.5 (100.0–123.0)	106.8 (90.0–118.6)	0.131
Platelet, median (IQR), * 10^9/L	196.0 (146.0–238.0)	152.0 (94.5–233.3)		< 0.001	183.5 (144.3–231.3)	166.0 (126.0–231.0)	0.136
D-Dimer, median (IQR), mg/L	0.5 (0.3–1.2)	0.8 (0.5–1.9)		0.030	0.6 (0.3–1.7)	0.8 (0.5–1.9)	0.687
RVD, median (IQR), mm	22.0 (18.0–29.0)	22.0 (17.1–29.0)		0.769	21.0 (18.0–25.0)	21.0 (18.0–25.0)	0.750
LVDs, median (IQR), mm	38.0 (29.0–46.0)	38.0 (32.5–50.5)		0.065	39.0 (32.0–45.5)	37.0 (24.8–44.5)	0.464
mPAD, median (IQR), mm	23.0 (20.0–27.8)	22.0 (21.0–26.0)		0.852	23.0 (21.0–26.0)	23.0 (23.0–28.0)	0.053
AOD, median (IQR), mm	21.8 (20.0–26.0)	25.0 (20.0–26.0)		0.234	25.0 (20.0–25.0)	25.0 (22.8–25.0)	0.230
EF, median (IQR), %	63.0 (60.0–66.0)	61.0 (57.0–64.0)		0.003	63.0 (61.0–67.0)	62.0 (56.0–66.0)	0.015
PASP, median (IQR), mm Hg	50.0 (42.0–71.5)	75.0 (50.0–100.0)		< 0.001	42.5 (37.0–56.3)	62.0 (41.0–90.0)	< 0.001

Two-tailed *P* values < 0.05 were considered statistically significant

*APTT* activated partial thromboplastin time, *AOD* aortic diameter, *C-section* Caesarean section, *EF* ejection fractions, *HTN* hypertension, *IQR* interquartile ratio, *LVDs* left ventricular systolic diameter, *mPAD* mean pulmonary artery diameter, *NYHA* New York Heart Association, *NT-proBNP* N-terminal pro-brain natriuretic peptide, *PASP* pulmonary artery systolic pressure, *PH* pulmonary hypertension, *RBC* red blood cells, *RVD* right ventricular diameter

**Table 2:** Demographic and clinical characteristics of patients in the follow-up and external validation sets

Variable	Follow-up set (n = 355)	External validation set (n = 221)
Age, median (IQR), year	28.0 (25.0–32.0)	30.0 (27.5–34.0)
follow-up duration, median (IQR), month	64.0 (42.0–83.0)	22.0 (14.5–27.0)
Death or heart failure, No. (%)		
No	266 (74.9)	183 (82.8)
Yes	89 (25.1)	38 (17.2)
Death, No. (%)		
No	343 (96.6)	217 (98.2)
Yes	12 (3.4)	4 (1.8)
Heart failure, No. (%)		
No	278 (78.3)	187 (84.6)
Yes	77 (21.7)	34 (15.4)
Mild preeclampsia, No. (%)		
No	348 (98.0)	210 (95.0)
Yes	7 (2.0)	11 (5.0)
Severe preeclampsia, No. (%)		
No	308 (86.8)	176 (79.6)
Yes	47 (13.2)	45 (20.4)
Eclampsia with pregnancy/delivery, No. (%)		
No	318 (89.6)	194 (87.8)
Yes	37 (10.4)	27 (12.2)
Eclampsia with pre-existing HTN, No. (%)		
No	344 (96.9)	212 (95.9)
Yes	11 (3.1)	9 (4.1)
Postpartum haemorrhage, No. (%)		
No	341 (96.1)	201 (91.0)
Yes	14 (3.9)	20 (9.0)
Multiple pregnancies, No. (%)		
No	339 (95.5)	192 (86.9)
Yes	16 (4.5)	29 (13.1)
Premature rupture of membranes, No. (%)		
No	336 (95.5)	205 (92.8)
Yes	16 (4.5)	16 (7.2)
Type I respiratory failure, No. (%)		
No	331 (93.2)	207 (93.7)
Yes	24 (6.8)	14 (6.3)
Type II respiratory failure, No. (%)		
No	353 (99.4)	219 (99.1)
Yes	2 (0.6)	2 (0.9)
Arrhythmia, No. (%)		
No	220 (62.0)	139 (62.9)
Yes	135 (38.0)	82 (37.1)
Patent ductus arteriosus, No. (%)		
No	343 (96.6)	209 (94.6)
Yes	12 (3.4)	12 (5.4)
Ventricular septal defect, No. (%)		
No	285 (80.3)	198 (89.6)
Yes	70 (19.7)	23 (10.4)
Atrial septal defect, No. (%)		
No	257 (72.4)	182 (82.4)
Yes	98 (27.6)	39 (17.6)
Pulmonary embolism, No. (%)		
No	347 (97.7)	220 (99.5)
Yes	8 (2.3)	1 (0.5)
Endocarditis, No. (%)		
No	354 (99.7)	220 (99.5)
Yes	1 (0.3)	1 (0.5)
Myocardiopathy, No. (%)		
No	340 (95.8)	219 (99.1)
Yes	15 (4.2)	2 (0.9)
Rheumatic heart disease, No. (%)		
No	326 (91.8)	212 (95.9)
Yes	29 (8.2)	9 (4.1)
Congenital heart disease, No. (%)		
No	150 (42.3)	134 (60.6)
Yes	205 (57.7)	87 (39.4)
Eisenmenger syndrome, No. (%)		
No	321 (90.4)	210 (95.0)
Yes	34 (9.6)	11 (5.0)
Gestational diabetes mellitus, No. (%)		
No	318 (89.6)	194 (87.8)
Yes	37 (10.4)	27 (12.2)
Infection, No. (%)		
No	313 (88.2)	170 (87.8)
Yes	42 (11.8)	51 (12.2)
Systemic lupus erythematosus, No. (%)		
No	343 (96.6)	211 (95.5)
Yes	12 (3.4)	10 (4.5)
Liver insufficiency, No. (%)		
No	349 (98.3)	209 (94.6)
Yes	6 (1.7)	12 (5.4)
Left to right shunt, No. (%)		
No	344 (96.9)	209 (94.6)
Yes	11 (3.1)	12 (5.4)
Right-to-left shunt, No. (%)		
No	351 (98.9)	217 (98.2)
Yes	4 (1.1)	4 (1.8)
Premature delivery, No. (%)		
No	152 (42.8)	87 (39.4)
Yes	152 (42.8)	107 (48.4)
PH classification, No. (%)		
Group 1	294 (82.8)	191 (86.4)
Group 2	50 (14.1)	18 (8.1)
Group 3	2 (0.6)	2 (0.9)
Group 4	2 (0.6)	0 (0.0)
Group 5	7 (2.0)	10 (4.5)
Pregnancy outcome, No. (%)		
Termination of pregnancy	51 (14.4)	26 (11.8)
Vaginal delivery	26 (7.3)	20 (9.0)
Spinal and/or epidural for C-section	240 (67.6)	164 (74.2)
General anaesthesia for C-section	38 (10.7)	11 (5.0)
Cardiac surgery, No. (%)		
No	339 (95.5)	204 (92.3)
Repair of heart defect	16 (4.5)	17 (7.7)
NYHA functional class, No. (%)		
I/II	203 (57.2)	155 (70.1)
III	91 (25.6)	39 (17.6)
IV	61 (17.2)	27 (12.2)
NT-proBNP, No. (%), ng/L		
< 1400	268 (75.5)	177 (80.1)
≥ 1400	87 (24.5)	44 (19.9)
Gestation times, median (IQR), times	2.0 (1.0–3.0)	2.0 (1.0–3.0)
Parity, median (IQR), times	1.0 (1.0–2.0)	1.0 (1.0–2.0)
Troponin, median (IQR), ng/mL	0.01 (0.00–0.03)	0.01 (0.00–0.15)
Prothrombin time, median (IQR), s	10.1 (9.5–10.8)	10.4 (9.7–11.2)
APTT, median (IQR), s	29.2 (26.8–32.4)	27.9 (26.1–30.7)
Thrombin time, median (IQR), s	13.8 (12.7–15.9)	14.3 (13.1–15.6)
Fibrinogen, median (IQR), g/L	3.7 (3.1–4.3)	3.8 (3.2–4.3)
RBC, median (IQR), × 10^12^/L	3.9 (3.5–4.3)	3.8 (3.4–4.2)
Haemoglobin, median (IQR), g/L	112.0 (101.0–124.0)	111.5 (100.0–123.0)
Platelet, median (IQR), × 10^9^/L	190.0 (129.8–237.0)	179.0 (137.0–228.0)
D-Dimer, median (IQR), mg/L	0.7 (0.4–1.3)	0.6 (0.3–1.6)
RVD, median (IQR), mm	22.0 (18.0–29.0)	20.0 (18.0–24.0)
LVDs, median (IQR), mm	38.0 (30.0–46.0)	40.0 (33.0–46.0)
mPAD, median (IQR), mm	23.0 (21.0–27.0)	23.0 (21.0–26.0)
AOD, median (IQR), mm	22.0 (20.0–26.0)	25.0 (20.0–25.0)
EF, median (IQR), %	63.0 (60.0–65.0)	63.0 (60.0–66.0)
PASP, median (IQR), mm Hg	53.0 (43.0–79.0)	44.0 (37.0–65.0)

Two-tailed *P* values < 0.05 were considered statistically significant

*APTT* activated partial thromboplastin time, *AOD* aortic diameter, *C-section* Caesarean section, *EF* ejection fractions, *HR* hazard ratio, *HTN* hypertension, *IQR* interquartile ratio, *LVDs* left ventricular systolic diameter, *mPAD* mean pulmonary artery diameter, *NYHA* New York Heart Association, *NT-proBNP* N-terminal pro-brain natriuretic peptide, *PASP* pulmonary artery systolic pressure, *PH* pulmonary hypertension, *RBC* red blood cells, *RVD* right ventricular diameter

### Nomogram development and validation (maternal mortality or HF)

**Fig. 1:**
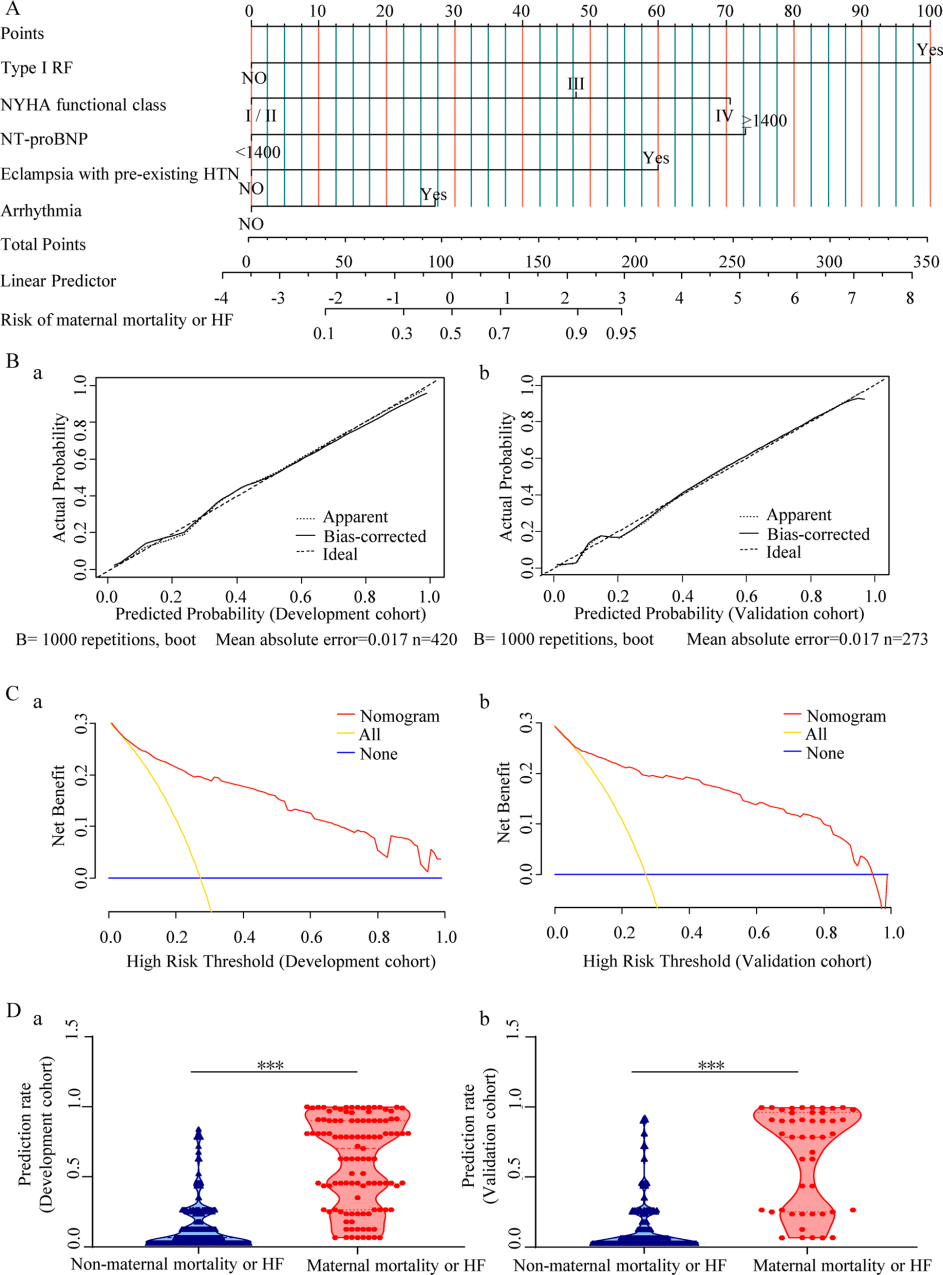
Nomogram development and validation (Maternal mortality or heart failure). **A** The nomogram incorporates five variables, with points allocated according to the scale for each variable. A total score was awarded from the sum of the individual scores, and used to calculate the predicted probability of maternal mortality or heart failure. **B (a, b)** Calibration curves for the nomogram in the Development (**a**) and Validation (**b**) cohorts. The calibration plot illustrates the accuracy of the original prediction (“Apparent”: light dotted line) and bootstrap models (“Bias-corrected”: solid line) in predicting the probability of maternal mortality or heart failure. **C (a, b)** Decision curve analysis for the nomogram in the Development (**a**) and Validation (**b**) cohorts. The y-axis indicates the net benefit, which is the sum of the benefits (true positives) minus harm (false positives). The x-axis indicates the threshold probability. The red line represents the nomogram net benefit. The yellow and blue lines represent the hypotheses that all or no patients experienced maternal mortality or heart failure, respectively. **D (a, b)** Violin plot analysis compared the distribution of risk prediction probabilities for patients experiencing maternal mortality or HF versus those without maternal death or HF in the Development (**a**) and Validation (**b**) cohorts. Demonstration of a violin plot and the depicted data. The three lines within the plot show the 1st and 3rd quartiles and the median of the dataset; the violin body width indicates the density of data along the y-axis. The violin edges represent the minimum and maximum values of the dataset. *HTN* hypertension, *HF* heart failure, *NT-proBNP* N-terminal pro-brain natriuretic peptide, *NYHA* New York Heart Association, *RF* respiratory failure

We constructed a nomogram to predict the risks of maternal mortality or HF in the Development cohort (n = 420). Multivariate logistic analysis identified the following independent predictors: type I respiratory failure (RF), New York Heart Association (NYHA) functional class, N-terminal pro-brain natriuretic peptide (NT-proBNP) ≥ 1400 ng/L, arrhythmia, and eclampsia with pre-existing hypertension (HTN) (Additional file 1: Fig. S1A). These were presented in nomogram form (Fig. 1A). For application of the dynamic nomogram, a score was awarded to correspond with each variable, and the sum of scores was recorded as the total score. The total score corresponded with the predicted risk of maternal mortality or HF in patients with PH (Additional file 1: Fig. S1B).

To evaluate the nomogram’s efficacy and its potential for clinical use, we performed internal and external validation. The bootstrap-corrected C-indexes from the Development and Validation cohorts were 0.892 (95% CI: 0.818–0.966) and 0.877 (95% CI: 0.757–0.996), respectively; this indicated excellent discriminative ability. Furthermore, the calibration plot showed that the predicted probabilities were close to actual observations in both cohorts (Fig. 1B, a–b). The results of the Hosmer–Lemeshow test (χ^2^ = 43.225; *P* = 0.873) indicated a good nomogram fit. Moreover, the AUC of the nomogram’s ROC curve for both cohorts also had good discrimination ability. As shown in Additional file 1: Fig. S1C, the AUC of the training cohort was 0.892 (95% CI: 0.855–0.929); this was verified in the Validation cohort with an AUC of 0.899 (95% CI: 0.843–0.955). The DCA curve demonstrated that the optional nomogram threshold probability was high and relatively safe in both cohorts, with a high net benefit (Fig. 1C, a–b). The nomogram was further used to calculate the probability of maternal mortality or HF for all patients. Violin plot analysis showed that the predicted risks for maternal mortality or HF were markedly lower in the surviving patients without HF in both cohorts (the nomogram predicted probabilities of mortality/HF as 0.612 ± 0.030 vs. survivors/no HF as 0.140 ± 0.010, *P* < 0.001 in the Development cohort and 0.645 ± 0.051 vs 0.137 ± 0.012, respectively, *P* < 0.001 in the Validation cohort) (Fig. 1D, a–b).

### Nomogram development and validation (overall survival)

**Fig. 2:**
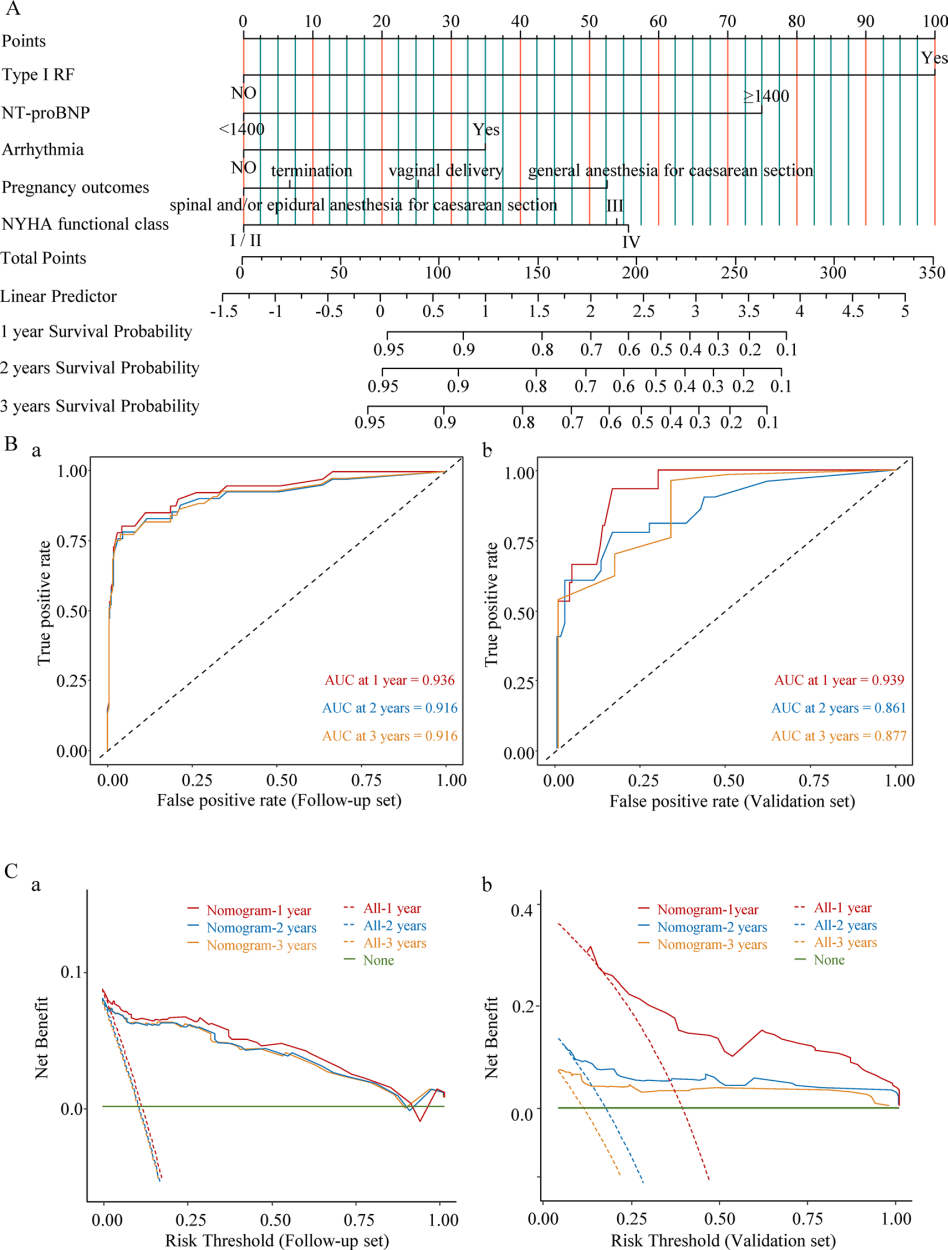
Nomogram development and validation (Overall survival). **A:** The nomogram incorporates five variables, with points allocated according to the scale for each variable. A total score was awarded from the sum of the individual scores, and used to calculate the predicted overall survival (OS) of pregnant patients with PH at 1, 2, and 3 years. **B (a, b):** ROC curve of the 1, 2, and 3-year survival predictions in the Follow-up (**a**) and (follow-up) Validation sets (**b**). The red, blue, and orange lines represent the AUC of the ROC curves for 1, 2, and 3 years, respectively. **C (a, b):** Decision curve analysis for the prognostic nomogram in the Follow-up (**a**) and (follow-up) Validation sets (**b**). The y-axis indicates the net benefit, which is the sum of the benefits (true positives) minus harm (false positives). The x-axis indicates the threshold probability. The red, blue, and orange lines represent the 1, 2, and 3-year survival benefits, respectively. *AUC* area under the curve, *NT-proBNP* N-terminal pro-brain natriuretic peptide, *NYHA* New York Heart Association, *PH* pulmonary hypertension, *RF* respiratory failure, *ROC* receiver operating characteristic

**Fig. 3:**
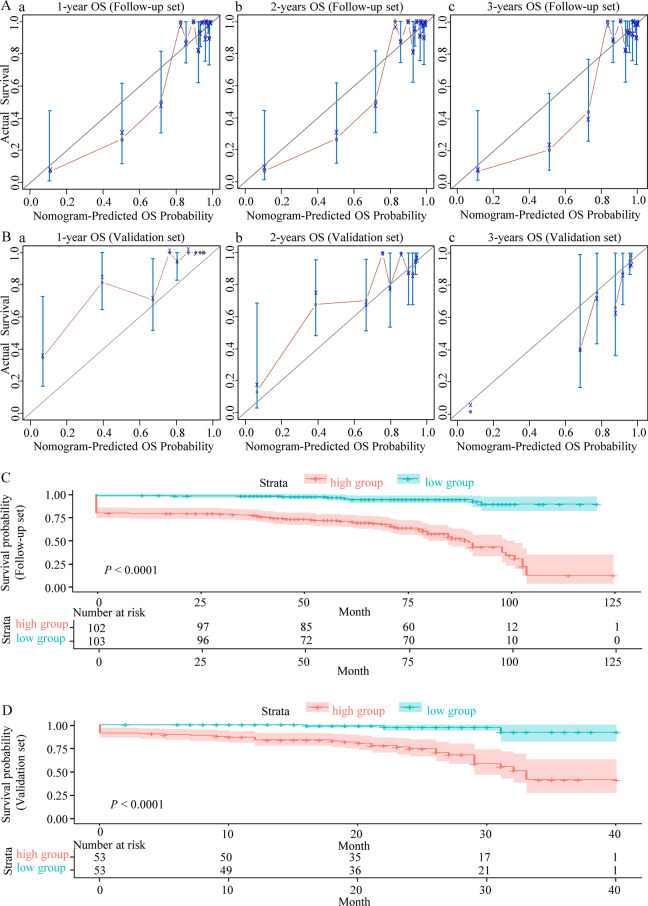
Calibration curves of the 1, 2, and 3-year overall survival and risk stratification. **A (a–c)** Calibration curves of the 1, 2, and 3-year OS for pregnant women with PH in the Follow-up set. **B (a–c)** Calibration curves of the 1, 2, and 3-year OS for pregnant women with PH in the Validation set. The light blue line indicates the ideal reference line where predicted probabilities would match the observed survival rates. The red dots are calculated by bootstrapping (resample: 1000) and represent the nomogram’s performance. The closer the solid red line is to the light blue line, the more accurately the model predicts survival. **C** Kaplan–Meier OS curves for the low-risk and high-risk pregnant women with PH stratified by the prognostic nomogram in the Follow-up set. According to the median cut-off value, samples were divided into high-risk and low-risk groups. **D** Kaplan–Meier OS curves for the low-risk and high-risk pregnant women with PH stratified by the prognostic nomogram in the Validation set. *OS* overall survival, *PH* pulmonary hypertension

In the Follow-up set, the independent prognostic factors type I RF, arrhythmia, general anaesthesia for caesarean sections (C-section), NYHA functional class, and NT-proBNP ≥ 1400 ng/L (Additional file 1: Fig. S2A) were used to create a prognostic nomogram for OS (Fig. 2A). In the nomogram and dynamic nomogram applications, each variable was assigned a value, the sum of which represents the total score. The predicted 1, 2, and 3-year OS rates corresponded with the overall score (Additional file 1: Fig. S2B). We then used a time-dependent AUC to verify the predictive capacity of the prognostic model. In the Follow-up set, the OS predictions at 1, 2, and 3 years were 0.936, 0.916, and 0.916, respectively (Fig. 2B.a), while those in the Validation set were 0.939, 0.861, and 0.877, respectively (Fig. 2B.b). These results indicated satisfactory predictive performance. On DCA, the nomogram offered a net benefit over the ‘treat-all’ or ‘treat-none’ strategy for both sets (Fig. 2C, a–b). The calibration curves of the nomogram demonstrated good correlation between the 1, 2, and 3-year predicted and actual survival probabilities for both sets (Fig. 3A, B).

### Risk stratification based on the prognostic nomogram

Risk stratification was performed based on the total scores of the prognostic nomogram. Patients in the Follow-up and Validation sets were divided into low and high-risk groups, with the median risk score representing the cut-off value. Kaplan–Meier analysis was used to explore survival differences using the log-rank test; the OS curves demonstrated excellent discrimination between the two risk groups in the different sets. Survival time was significantly shorter in the high-risk than low-risk groups for all sets (Fig. 3C, D).

### Nomogram development and validation (Foetal/neonatal adverse events)

**Fig. 4:**
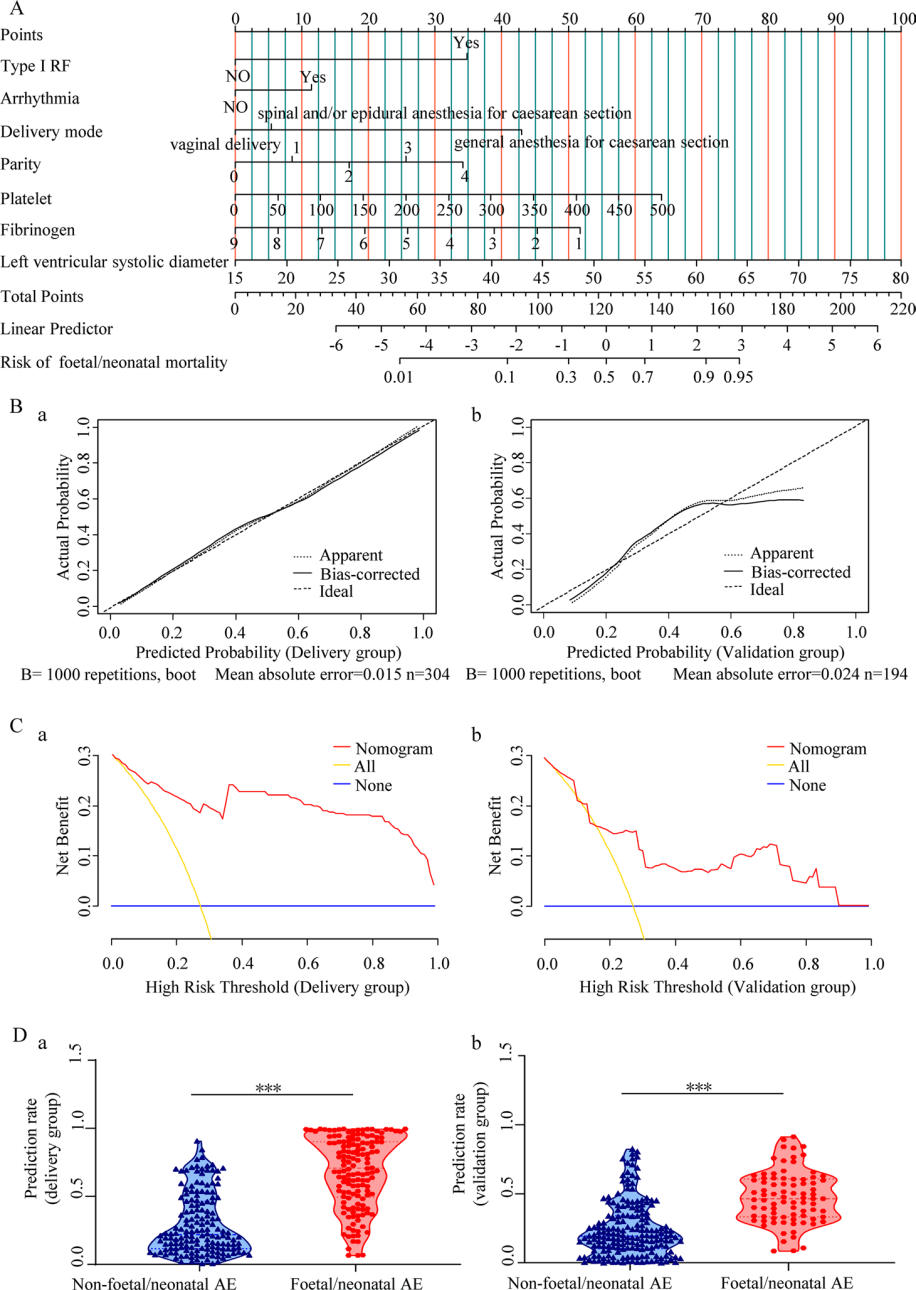
Nomogram development and validation (Adverse foetal/neonatal events). **A** The nomogram incorporates seven variables, with points allocated according to the scale for each variable. A total score was awarded from the sum of the individual scores, and used to calculate the predicted probability of adverse foetal/neonatal events. **B (a, b)** Calibration curves for the nomogram in the Delivery (**a**) and Validation (**b**) groups. The calibration plot illustrates the accuracy of the original prediction (“Apparent”: light dotted line) and bootstrap models (“Bias-corrected”: solid line) in predicting the probability of adverse foetal/neonatal events. **C (a, b)** Decision curve analysis for the nomogram in the Delivery (**a**) and Validation (**b**) groups. The y-axis indicates the net benefit, which is the sum of the benefits (true positives) minus harm (false positives). The x-axis indicates the threshold probability. The red line represents the net nomogram benefit. The yellow and blue lines represent the hypotheses that all and no patients had adverse foetal/neonatal events, respectively. **D (a, b)** Violin plot analysis compared the distribution of risk prediction probabilities for those with adverse foetal/neonatal events versus those without in the Delivery (**a**) and Validation (**b**) groups. The predicted risk for those with adverse foetal/neonatal events was markedly higher than for those without adverse events in both groups. Demonstration of a violin plot and the depicted data. The three lines within the plot show the 1st and 3rd quartiles and the median of the dataset; the violin body width indicates the density of data along the y-axis. The violin edges represent the minimum and maximum dataset values. *AE* adverse event, *RF* respiratory failure

For the Delivery group, multivariate logistic regression analysis identified seven variables (type I RF, NT-proBNP ≥ 1400 ng/L, arrhythmia, general anaesthesia for C-section, parity, platelets, fibrinogen, and left ventricular systolic diameter) as independent predictors for foetal/neonatal adverse clinical events (Additional file 1: Fig. S4A). These were used to build another nomogram (Fig. 4A). On ROC curves, fibrinogen reduction to ≤ 2.00 g/L, platelet count > 245 × 10^9^/L, parity > 2, and maternal left ventricular systolic diameter $\ge$ 35 mm were significant thresholds for predicting foetal/neonatal adverse events. The nomogram and dynamic nomogram applications are depicted in Additional file 1: Fig. S4B.

Internal validation was performed using bootstrapping resampling for 1000 repetitions, and the C-index value was 0.854 (95% CI: 0.770–0.938) in the Delivery group and 0.764 (95% CI: 0.639–0.889) in the Validation group. The AUC of the Delivery and Validation groups were 0.854 (95% CI: 0.812–0.896) and 0.764 (95% CI: 0.701–0.828), respectively (Additional file 1: Fig. S4C). Notably, the calibration plot showed that the predicted probabilities were close to the actual observed outcomes for the Delivery (Fig. 4B.a) and Validation groups (Fig. 4B.b). Results of the Hosmer–Lemeshow test of the model in the Delivery group (χ^2^ = − 0.544, *P* = 0.586) indicated a good nomogram fit. Moreover, DCA curves revealed that the nomogram may better predict the risk of foetal/neonatal adverse events than the ‘treat-all’ and ‘treat-none’ schemes, as it added more net benefits for both groups (Fig. 4C). The nomogram constructed by logistic regression was used to calculate the probability of foetal/neonatal adverse events for all pregnant patients with PH. Nomogram scores of the foetal/neonatal adverse events patients were markedly higher than those of the patients without foetal/neonatal adverse events (the nomogram predicted probabilities of 0.678 ± 0.021 vs 0.298 ± 0.018, *P* < 0.001 in the Delivery group and 0.477 ± 0.021 vs 0.242 ± 0.014, *P* < 0.001 in the Validation group, respectively) (Fig. 4D). This implies that this model may accurately predict a patient's risk probability of having foetal/neonatal adverse events.

### Implementation of the web server

We designed three dynamic nomograms using practical online applications; each incorporated an independent predictor. The online application for predicting maternal mortality or HF is available at https://ph-666.shinyapps.io/maternal-D/ (Additional file 1: Fig. S1D), for maternal prognosis at https://ph-666.shinyapps.io/COX-pregnant/ (Additional file 1: Fig. S3A–C), and for neonatal adverse events at https://ph-666.shinyapps.io/AE-fetal/ (Additional file 1: Fig. S4D).

## Discussion

### Principal findings

Using the parameters of type I RF, NYHA functional class, NT-proBNP $\ge$ 1400 ng/L, eclampsia with pre-existing HTN, and arrhythmia, this nomogram is a useful screening tool for patients at high risk of maternal mortality or HF. This personalised approach also represents a dynamic online tool with a user-friendly digital interface (https://ph-666.shinyapps.io/maternal-D/). Most maternal deaths occur during the first week postpartum due to postdelivery hemodynamic changes. Moreover, five variables (type I RF, arrhythmia, NT-proBNP $\ge$ 1400 ng/L, pregnancy outcomes, and NYHA functional class) were incorporated into a nomogram to predict OS and monitor PH condition over time. To enable easy clinical implementation, we developed a dynamic online nomogram application (https://ph-666.shinyapps.io/COX-pregnant/). Pregnancy in women with PH has long been regarded as a high risk for both maternal and foetal/neonatal complications [7]. We found that type I RF, NT-proBNP $\ge$ 1400 ng/L, arrhythmia, delivery mode, parity, platelet, fibrinogen, and left ventricular systolic diameter were independently associated with increased adverse foetal/neonatal events. Further, these online dynamic nomograms are available at https://ph-666.shinyapps.io/AE-fetal/. Our online nomograms ascertained the probabilities of maternal mortality, survival, and the foetal/neonatal adverse events when the corresponding clinical factors were input.

### Results in the context of what is known

PH affects pulmonary vasculature and the heart, and pregnancy places a significant burden on the cardiovascular system. Like other studies [2], we found that maternal mortality or HF were associated with NYHA functional class. Pregnant patients with NYHA functional class IV had poorer pregnancy outcomes. NT-proBNP is also recommended by international guidelines for risk assessment in patients with PH [27]. Moreover, since pregnancy is a hypermetabolic state, oxygen consumption increases by approximately 20% [28]. Increased minute ventilation leads to respiratory alkalosis, increased arterial oxygen tension, and dyspnoea. In our study, lower oxygen partial pressure was associated with maternal and foetal/neonatal complications [6]. Pregnant patients with hypoxia can also experience placental hypoperfusion, which can affect foetal growth and lead to perinatal complications [29].

Consistent with previous studies, maternal arrhythmias were found to be associated with an increase in maternal cardiac events and foetal/neonatal adverse outcomes [30, 31]. In particular, hypertensive disorders of pregnancy are associated with higher rates of maternal mortality [32]. Our results are consistent with recent studies where the administration of general anaesthesia during C-section is associated with an increased incidence of adverse maternal [1, 33], and foetal/neonatal outcomes [34, 35]. This may be related to increased intrathoracic and pulmonary artery pressure and thus, reduced venous return from endotracheal intubation and positive pressure ventilation [2]. The latest guidelines from the ESC reflect existing evidence that planned caesarean or vaginal deliveries carry lower risk [36]. Our study found lower risks of death or HF in women who delivered via C-section with spinal or epidural anaesthesia than via vaginal delivery, which is consistent with other literature favouring or even recommending planned C-sections [1, 2, 37]. Vaginal delivery can cause pain and increase thoracic pressure, which may reduce venous reflux [38]. In contrast, C-section provides a more regulated delivery environment [39]. It avoids long labour and allows for careful preparation of anaesthesia, haemodynamic optimisation, and contingency planning [1, 40]. However, more research is needed to determine the optimal delivery method. The current guidelines recommend strict contraception for patients with PH, and early pregnancy termination [3]; however, termination has also been associated with high maternal risks [5]; this was consistent with our findings. Our results suggested that a fibrinogen reduction to ≤ 2.00 g/L, platelet count > 245 × 10^9^/L, parity > 2, and maternal left ventricular systolic diameter $\ge$ 35 mm were the significant thresholds for predicting foetal/neonatal adverse events. Fibrinogen level before delivery may be a good predictor of placental abruption [41]. Decreased fibrinogen level may be associated with the degree of sub-placental hematoma formation, resulting in acute/chronic foetal acidaemia and increased intrauterine growth restriction [42]. Maternal platelet count has been used as an early predictor of neonatal respiratory distress and adverse foetal/neonatal outcomes [43]. Consistent with previous studies, parity was associated with growth restriction and mortality in neonates [44]. Interestingly, left ventricular mass has been shown to have a strong heritability component for cardiac features [45, 46].

### Clinical implications

This was the first study to develop and validate a practical nomogram to predict maternal mortality or HF, OS, and adverse foetal/neonatal events in patients with PH. The nomogram was based on large-scale data analysis, and its intuitive icon model was convenient for clinical application. It can also be developed into an online application for intelligent patient management as well as auxiliary diagnosis and treatment. Nomograms have achieved good clinical application results in the fields of cancer, cardiovascular, and other diseases. The nomograms constructed in this study had good application results, including primary medical institutions' preliminary judgement of the prognostic risk of patients with pregnant women with PH and early identification of high-risk patients for early referral, evaluation, and treatment. It can also provide clinicians with accurate prediction tools for individual mortality, heart failure, overall survival, and adverse foetal/neonatal clinical events. The online tools developed based on the nomograms can be used for the self-management by pregnant patients with PH through an intelligent communication terminal operation programme and can be integrated with a hospital case management system for artificial intelligence-assisted diagnosis. The machine learning and feedback optimisation in the later application process will further improve the efficiency and accuracy of clinical prediction.

### Strengths and limitations of the study

This study has some limitations, including limited cohort size and potential selection bias. Furthermore, data interpretation revealed some limitations to echocardiography as a diagnostic method: although RHC is the gold standard for PH diagnosis, radiation exposure may lead to foetal teratogenicity, carcinogenicity, or mutation. Spontaneous abortion, growth restriction, and intellectual disabilities may occur with high exposure levels. Regardless of the dose, the cancer risk also increases; therefore, the Swan–Ganz catheter is not recommended for routine perinatal monitoring [47]. Another limitation is that the patient populations of the Development and external Validation cohorts differed, and the sample size of the external Validation cohort was relatively small. Although the external validation proves a good calibration, its validation efficiency is limited. Lastly, a larger prospective multi-centre study of patients with PH is needed to determine the exact risks associated with pregnancy, the role of supportive care and late PH treatment, and prognostic factors for each subgroup.

## Conclusions

The nomograms created in this study may be used to accurately predict maternal mortality or HF, adverse foetal/neonatal outcomes, and survival in pregnant patients with PH. Additionally, these tools can guide more effective clinical decision-making. Our nomograms and accompanying software can assess individualised risks and anticipate OS more easily in pregnant patients with PH. The online nomogram software was shown to be a useful tool for the management of pregnant patients with PH for primary hospitals or community centres.

## Supplementary Information

**Additional file 1.** Supplementary materials for the prediction and prognosis of adverse maternal and foetal/neonatal outcomes in pulmonary hypertension.

## Data Availability

All data included in this study are available upon request through contact with the corresponding author.

## Abbreviations

AHA: American Heart Association
AOD: Aortic Diameter
APTT: Activated partial thromboplastin time
AUC: Area under the curve
CI: Confidence interval
C-index: Concordance index
C-section: Caesarean section
DCA: Decision curve analysis
EF: Ejection fraction
ESC: European Society of Cardiology
HF: Heart failure
HR: Hazard ratio
HTN: Hypertension
IQR: Interquartile ratio
LVD: Left ventricular (systolic) diameter
NT-proBNP: N-terminal pro-brain natriuretic peptide
NYHA: New York Heart Association
OS: Overall survival
PASP: Pulmonary artery systolic pressure
PH: Pulmonary hypertension
RBC: Red blood cells
RF: Respiratory failure
RHC: Right heart catheterisation
ROC: Receiver operating characteristic
RVD: Right ventricular diameter
SGA: Small for gestational age
