# Correction to: Prediction and prognosis of adverse maternal and foetal/neonatal outcomes in pulmonary hypertension: an observational study and nomogram construction

**DOI:** 10.1186/s12931-022-02306-0

**Published:** 2023-01-07

**Authors:** Yuqin Chen, Dansha Zhou, Mingmei Xiong, Xin Xi, Wenni Zhang, Ruifeng Zhang, Lishi Chen, Qian Jiang, Ning Lai, Xiang Li, Jieer Luo, Xuanyi Li, Weici Feng, Chuhui Gao, Jiyuan Chen, Xin Fu, Wei Hong, Mei Jiang, Kai Yang, Wenju Lu, Yiping Luo, Jun Zhang, Zhe Cheng, Chunli Liu, Jian Wang

**Affiliations:** 1 State Key Laboratory of Respiratory Diseases, Guangdong Key Laboratory of Vascular Diseases, National Clinical Research Centre for Respiratory Diseases, Guangzhou Institute of Respiratory Health, The First Affiliated Hospital of Guangzhou Medical University, 151 Yanjiang Road, Guangzhou, 510120 Guangdong People’s Republic of China; 2 The Third Affiliated Hospital of Guangzhou Medical University, Guangzhou, 510140 Guangdong People’s Republic of China; 3 Sleep Centre and Department of Respiratory Medicine, Beijing Anzhen Hospital of Capital Medical University, Beijing, 100029 People’s Republic of China; 4 Guangdong Women and Children’s Hospital, 521 Xingnan Avenue, Panyu District, Guangzhou, 511442 Guangdong People’s Republic of China; 5 Department of Respiratory Medicine, Zhongda Hospital of Southeast University, Nanjing, 210009 People’s Republic of China; 6 GMU-GIBH Joint School of Life Sciences, Guangzhou Medical University, Guangzhou, Guangdong People’s Republic of China; 7 Pulmonary and Critical Care Medicine, The First Affiliated Hospital of Zhengzhou University, Zhengzhou, 450052 Henan People’s Republic of China; 8 Section of Physiology, Division of Pulmonary, Critical Care and Sleep Medicine, University of California, San Diego, La Jolla, CA USA; 9 Department of Obstetrics and Gynecology of Beijing Anzhen Hospital, Capital Medical University, No.2 Anzhen Road, 100029 Beijing, People’s Republic of China

**Correction to** **:** **Respiratory Research (2022) 23:314** **https://doi.org/10.1186/s12931-022-02235-y**

Following publication of the original article [1], the authors identified an error in Fig. 3. The correct version of Fig. 3 is given.

**Fig. 3:**
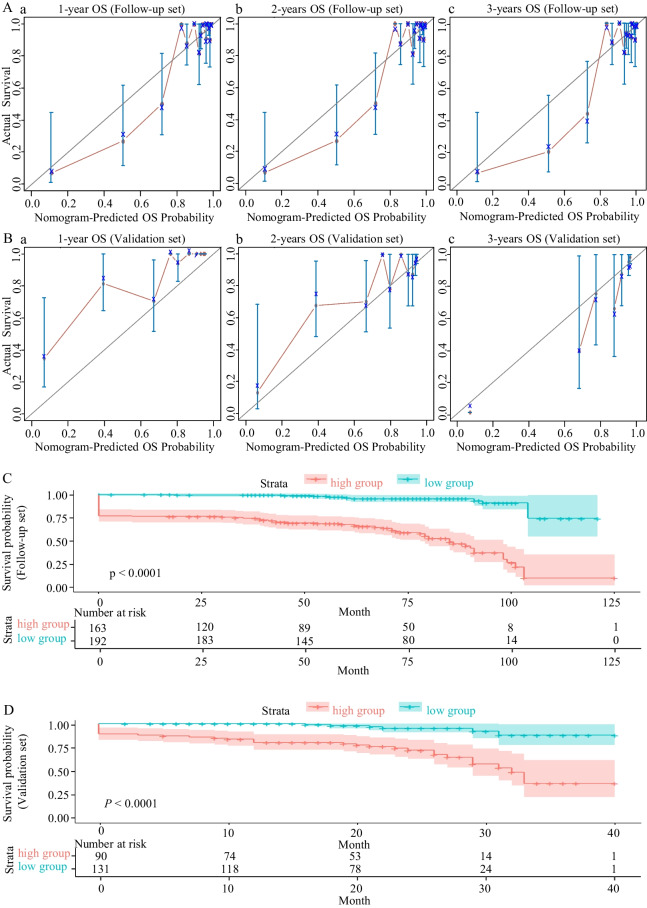
Calibration curves of the 1, 2, and 3-year overall survival and risk stratification. **A (a–c)** Calibration curves of the 1, 2, and 3-year OS for pregnant women with PH in the Follow-up set. **B (a–c)** Calibration curves of the 1, 2, and 3-year OS for pregnant women with PH in the Validation set. The light blue line indicates the ideal reference line where predicted probabilities would match the observed survival rates. The red dots are calculated by bootstrapping (resample: 1000) and represent the nomogram’s performance. The closer the solid red line is to the light blue line, the more accurately the model predicts survival. **C** Kaplan–Meier OS curves for the low-risk and high-risk pregnant women with PH stratified by the prognostic nomogram in the Follow-up set. According to the median cut-off value, samples were divided into high-risk and low-risk groups. **D** Kaplan–Meier OS curves for the low-risk and high-risk pregnant women with PH stratified by the prognostic nomogram in the Validation set. *OS* overall survival, *PH* pulmonary hypertension

The original article has been corrected.
