# Supplementary material for: Prediction and prognosis of adverse maternal and foetal/neonatal outcomes in pulmonary hypertension: an observational study and nomogram construction
Source: Respir Res. 2022 Nov 15;23:314. doi: 10.1186/s12931-022-02235-y (PMC9663284; doi:10.1186/s12931-022-02235-y)
Supplement: Supplementary file 1 — Additional file 1. Supplementary materials for the prediction and prognosis of adverse maternal and foetal/neonatal outcomes in pulmonary hypertension. [file 12931_2022_2235_MOESM1_ESM.docx]

**Supplementary materials**

**Prediction and prognosis of adverse maternal and foetal/neonatal outcomes in pulmonary hypertension: An observational study and nomogram construction**

Yuqin Chen et al.

**Corresponding author:**

**Jian Wang, PhD** (Email: jiw037@health.ucsd.edu)

**Chunli Liu, PhD** (Email: chunli@gird.cn)

**Zhe Cheng, PhD** (Email: fccchengzhe@zzu.edu.cn)

**Jun Zhang, PhD** (Email: drzhangj@outlook.com)

**Yiping Luo, M.D** (Email: doctorluoyiping@126.com)

State Key Laboratory of Respiratory Diseases

Guangzhou Institute of Respiratory Health

The First Affiliated Hospital of Guangzhou Medical University

151 Yanjiang Road, Guangzhou, Guangdong, 510120

People’s Republic of China

Tel: 86-20-83205097

Fax: 86-20- 83205074

**Materials and Methods**

**Patient characteristics and outcome measures**

The following data were collected: age; parity; previous interventions; New York Heart Association (NYHA) functional class; coagulation function tests; other biochemical examinations (first examination after termination or delivery); Pulmonary hypertension (PH)-targeted medication; cardiac events such as heart failure (HF) or arrhythmia; obstetric events such as diabetes mellitus, multiple pregnancies, eclampsia status (eclampsia complication > severe pre-eclampsia > mild pre-eclampsia > pre-eclampsia/eclampsia with pre-existing hypertension); postpartum hemorrhage; obstetric pulmonary embolism; delivery mode; aesthetic management; and postpartum follow-up evaluation. Postpartum hemorrhage was defined as increased blood loss (>500 ml after vaginal delivery or >1000 ml after caesarean delivery) both immediately and within 24 h of delivery [1]. Multiple imputation techniques have been developed to recover the information that would otherwise be lost when excluding observations with missing data and to help minimize bias [2].

**Statistical analysis**

Statistical analyses were performed using SPSS (version 26.0; IBM Corporation, Armonk, NY, US) and R software (version 4.0.2; R Foundation for Statistical Computing, Vienna, Austria). Categorical variables are presented as numbers and percentages, and normally distributed data for continuous variables as mean ± standard deviation; otherwise, median and interquartile range (IQR) are shown.

Based on the Cox regression nomogram used to calculate the risk score for all patients, we divided pregnant women with PH into high-risk and low-risk groups. The median risk score was used as the cut-off value, and Kaplan–Meier analysis was used to explore differences in survival probabilities of the different risk groups. Finally, all nomograms were constructed into a user-friendly web server that facilitated the practical clinical application of the nomograms. For all analyses, *P* < 0.05 was considered statistically significant. Odds ratios (ORs) and 95% confidence intervals (CIs) were calculated for summary statistics, and forest plots were generated to visualize the ORs and 95% CIs of the potential baseline predictive covariates.

**References**

1. Drenthen W, Boersma E, Balci A, Moons P, Roos-Hesselink JW, Mulder BJ, Vliegen HW, van Dijk AP, Voors AA, Yap SC, et al: Predictors of pregnancy complications in women with congenital heart disease. *Eur Heart J* 2010, 31:2124-2132.<https://doi.org/10.1093/eurheartj/ehq200>

2. Blazek K, van Zwieten A, Saglimbene V, Teixeira-Pinto A: A practical guide to multiple imputation of missing data in nephrology. *Kidney Int* 2021, 99:68-74.<https://doi.org/10.1016/j.kint.2020.07.035>

**SUPPLEMENTARY TABLES**

**Table S1 Univariate cox analyses on variables for the prediction of overall survival in the Follow-up and External validation sets**

| **Variable** | | **Follow-up set (n=355)** | | | **External validation set (n=221)** | | |
| --- | --- | --- | --- | --- | --- | --- | --- |
|  | **HR (95 % CI)** | | ***P.* Value** | **HR (95 % CI)** | | ***P.* Value** | |
| Age, median (IQR), year | 0.952 (0.896-1.011) | | 0.107 | 0.973 (0.911-1.038) | | 0.406 | |
| Mild preeclampsia, No. (%) |  | | 0.857 |  | | 0.655 | |
| Yes vs. No | 0.834 (0.115-6.058) | |  | 1.573 (0.215-11.503) | |  | |
| Severe preeclampsia, No. (%) |  | | 0.448 |  | | 0.602 | |
| Yes vs. No | 1.483 (0.532-4.167) | |  | 0.825 (0.400-1.701) | |  | |
| Eclampsia with pregnancy/delivery, No. (%) |  | | 0.810 |  | | 0.133 | |
| Yes vs. No | 1.134 (0.405-3.174) | |  | 2.977 (0.716-12.377) | |  | |
| Eclampsia with pre-existing HTN, No. (%) |  | | 0.554 |  | | 0.435 | |
| Yes vs. No | 0.651 (0.158-2.693) | |  | 21.277 (0.010-46024.331) | |  | |
| Postpartum haemorrhage, No. (%) |  | | 0.083 |  | | 0.358 | |
| Yes vs. No | 0.402 (0.144-1.125) | |  | 1.954 (0.469-8.142) | |  |  |
| Multiple pregnancies, No. (%) |  | | 0.506 |  | | 0.567 | |
| Yes vs. No | 1.961 (0.270-14.246) | |  | 1.354 (0.480-3.819) | |  | |
| Premature rupture of membranes, No. (%) |  | | 0.654 |  | | 1.000 | |
| Yes vs. No | 0.764 (0.236-2.472) | |  | 1.000 (0.239-4.183) | |  | |
| Type I respiratory failure, No. (%) |  | | $<$0.001 |  | | $<$0.001 | |
| Yes vs. No | 13.745 (7.933-23.815) | |  | 11.080 (5.539-22.165) | |  | |
| Type II respiratory failure, No. (%) |  | | 0.013 |  | | 0.255 | |
| Yes vs. No | 6.045 (1.460-25.026) | |  | 0.314 (0.043-2.311) | |  | |
| Arrhythmia, No. (%) |  | | $<$0.001 |  | | 0.233 | |
| Yes vs. No | 2.302 (1.511-3.508) | |  | 0.677 (0.357-1.2875) | |  | |
| Patent ductus arteriosus, No. (%) |  | | 0.005 |  | | 0.479 | |
| Yes vs. No | 3.862 (1.519-9.817) | |  | 0.652 (0.200-2.127) | |  | |
| Ventricular septal defect, No. (%) |  | | 0.342 |  | | 0.898 | |
| Yes vs. No | 1.394 (0.730-2.766) | |  | 0.934 (0.331-2.635) | |  | |
| Atrial septal defect, No. (%) |  | | 0.050 |  | | 0.117 | |
| Yes vs. No | 0.422 (0.178-1.000) | |  | 2.571 (0.791-8.364) | |  | |
| Pulmonary embolism, No. (%) |  | | 0.002 |  | | 0.833 | |
| Yes vs. No | 5.258 (1.877-14.724) | |  | 20.239 (0.000-2.620E+13) | |  | |
| Endocarditis, No. (%) |  | | 0.029 |  | | 0.022 | |
| Yes vs. No | 9.080 (1.248-66.068) | |  | 10.534 (1.409-78.741) | |  | |
| Myocardiopathy, No. (%) |  | | 0.534 |  | | 0.003 | |
| Yes vs. No | 0.533 (0.073-3.871) | |  | 9.136 (2.145-38.906) | |  | |
| Rheumatic heart disease, No. (%) |  | | 0.790 |  | | 0.632 | |
| Yes vs. No | 1.150 (0.411-3.218) | |  | 1.626 (0.230-2.449) | |  | |
| Congenital heart disease, No. (%) |  | | 0.726 |  | | 0.218 | |
| Yes vs. No | 1.115 (0.605-2.056) | |  | 1.555 (0.771-3.137) | |  | |
| Eisenmenger syndrome, No. (%) |  | | $<$0.001 |  | | 0.635 | |
| Yes vs. No | 4.170 (2.174-7.997) | |  | 0.751 (0.230-2.449) | |  | |
| Gestational diabetes mellitus, No. (%) |  | | 0.079 |  | | 0.316 | |
| Yes vs. No | 1.992 (0.924-4.294) | |  | 2.075 (0.498-8.644) | |  | |
| Infection, No. (%) |  | | 0.001 |  | | 0.018 | |
| Yes vs. No | 2.972 (1.524-5.793) | |  | 2.214 (1.144-4.287) | |  | |
| Systemic lupus erythematosus, No. (%) |  | | 0.035 |  | | 0.076 | |
| Yes vs. No | 3.033 (1.083-8.493) | |  | 0.339 (0.103-1.120) | |  | |
| Liver insufficiency, No. (%) |  | | 0.736 |  | | 0.454 | |
| Yes vs. No | 1.407 (0.194-10.225) | |  | 0.595 (0.142-2.502) | |  | |
| Left to right shunt, No. (%) |  | | 0.138 |  | | 0.479 | |
| Yes vs. No | 0.411 (0.127-1.329) | |  | 0.450 (0.061-3.306) | |  | |
| Right-to-left shunt, No. (%) |  | | 0.039 |  | | 0.433 | |
| Yes vs. No | 4.475 (1.081-18.520) | |  | 4.475 (1.081-18.520) | |  | |
| Premature delivery, No. (%) |  | | 0.320 |  | | 0.218 | |
| Yes vs. No | 1.258 (0.800-1.981) | |  | 0.631 (0.303-1.313) | |  | |
| PH classification, No. (%) |  | | 0.006 |  | | 0.891 | |
| Group 2 vs. Group 1 | 1.093 (0.457-2.615) | | 0.842 | 0.713 (0.170-2.992) | | 0.644 | |
| Group 3 vs. Group 1 | 0.000 (0.000-1.0012E+297) | | 0.977 | 0.000 (0.000-1.821E+282) | | 0.974 | |
| Group 4 vs. Group 1 | 9.931 (2.374-41.553) | | 0.002 | .. | | .. | |
| Group 5 vs. Group 1 | 4.106 (1.256-13.424) | | 0.019 | 1.445 (0.442-4.728) | | 0.542 | |
| Pregnancy outcome, No. (%) |  | | $<$0.001 |  | | 0.001 | |
| Termination vs. general anaesthesia for C-section | 0.332 (0.170-0.648) | | 0.001 | 0.585 (0.193-1.772) | | 0.343 | |
| Vaginal delivery vs. general anaesthesia for C-section | 0.234 (0.096-0.571) | | 0.001 | 0.393 (0.113-1.371) | | 0.143 | |
| spinal and/or epidural for C-section vs. general anaesthesia for C-section | 0.160 (0.097-0.265) | | $<$0.001 | 0.169 (0.062-0.458) | | $<$0.001 | |
| Cardiac surgery, No. (%) |  | | 0.500 |  | | 0.238 | |
| Repair of heart defect vs. No | 0.505 (0.070-3.673) | |  | 0.741 (0.451-1.219) | |  | |
| NYHA functional class, No. (%) |  | | $<$0.001 |  | | $<$0.001 | |
| III vs. I / II | 3.724 (2.075-6.684) | | $<$0.001 | 3.935 (1.669-9.280) | | 0.002 | |
| IV vs. I / II | 8.391 (4.873-14.447) | | $<$0.001 | 9.512 (4.449-20.340) | | $<$0.001 | |
| NT-proBNP, No. (%), ng/L |  | | $<$0.001 |  | | $<$0.001 | |
| $\geq$1400 vs. <1400 | 6.809 (4.385-10.573) | |  | 6.487 (3.403-12.367) | |  | |
| Gestation times, median (IQR), times | 0.782 (0.586-1.042) | | 0.094 | 0.907 (0.712-1.156) | | 0.430 | |
| Parity, median (IQR), times | 0.659 (0.407-1.067) | | 0.090 | 0.870 (0.571-1.326) | | 0.516 | |
| Troponin, median (IQR), ng/mL | 1.001 (1.000-1.001) | | 0.207 | 1.033 (1.000-1.068) | | 0.053 | |
| Prothrombin time, median (IQR), s | 1.017 (1.003-1.030) | | 0.014 | 1.121 (1.055-1.192) | | $<$0.001 | |
| APTT, median (IQR), s | 1.092 (1.054-1.131) | | $<$0.001 | 1.004 (0.962-1.134) | | 0.298 | |
| Thrombin time, median (IQR), s | 1.041 (1.015-1.067) | | 0.002 | 0.996 (0.847-1.171) | | 0.957 | |
| Fibrinogen, median (IQR), g/L | 0.738 (0.541-1.007) | | 0.055 | 1.137 (0.853-1.515) | | 0.382 | |
| RBC, median (IQR), * 10^12/L | 1.070 (1.006-1.027) | | 0.002 | 0.805 (0.505-1.282) | | 0.360 | |
| Haemoglobin, median (IQR), g/L | 1.001 (0.987-1.015) | | 0.926 | 0.991 (0.976-1.006) | | 0.230 | |
| Platelet, median (IQR), * 10^9/L | 0.985 (0.981-0.990) | | $<$0.001 | 0.996 (0.991-1.000) | | 0.074 | |
| D-Dimer, median (IQR), mg/L | 1.000 (1.000-1.000) | | 0.006 | 1.014 (0.905-1.136) | | 0.815 | |
| RVD, median (IQR), mm | 1.043 (0.998-1.090) | | 0.060 | 1.002 (0.943-1.066) | | 0.944 | |
| LVDs, median (IQR), mm | 0.973 (0.972-1.017) | | 0.224 | 0.974 (0.931-1.019) | | 0.255 | |
| mPAD, median (IQR), mm | 1.011 (0.951-1.075) | | 0.727 | 1.061 (0.999-1.128) | | 0.055 | |
| AOD, median (IQR), mm | 1.033 (0.960-1.111) | | 0.385 | 1.064 (0.976-1.150) | | 0.158 | |
| EF, median (IQR), % | 0.972 (0.938-1.007) | | 0.120 | 0.972 (0.937-1.008) | | 0.132 | |
| PASP, median (IQR), mm Hg | 1.027 (1.017-1.037) | | $<$0.001 | 1.019 (1.010-1.029) | | $<$0.001 | |

Two-tailed *P* values < 0.05 were considered statistically significant.

Abbreviations: APTT, activated partial thromboplastin time; AOD, aortic diameter; CI, confidence interval; C-section, Caesarean section; EF, Ejection Fractions; HR, hazard ratio; HTN, hypertension; IQR, Interquartile ratio; LVDs, left ventricular systolic diameter; mPAD, mean pulmonary artery diameter; NYHA, New York Heart Association; NT-proBNP, N-terminal pro-brain natriuretic peptide; PASP, pulmonary artery systolic pressure; PH, pulmonary hypertension; RBC, Red blood cells; RVD, right ventricular diameter.

**Table S2 Demographic and clinical characteristics of patients in the Delivery and External validation groups**

| **Variable** | | **Delivery group** | |  | | **External validation group** | | |  | |
| --- | --- | --- | --- | --- | --- | --- | --- | --- | --- | --- |
|  | **Non-death or HF (n = 158)** | | **Death or HF (n = 146)** | | ***P* Value** | | **Non-death or HF (n = 145)** | **Death or HF (n = 83)** | | ***P* Value** |
| Age, median (IQR), year | | 29.0 (25.8-33.0) | 28.0 (25.0-32.0) | | 0.331 | | 31.0 (28.0-34.5) | 29.0 (27.0-32.0) | | 0.024 |
| Mild preeclampsia, No. (%) | |  |  | | 0.228 | |  |  | | 0.070 |
| No | | 156 (98.7) | 141 (96.6) | |  | | 134 (92.4) | 82 (98.8) | |  |
| Yes | | 2 (1.3) | 5 (3.4) | |  | | 11 (7.6) | 1 (1.2) | |  |
| Severe preeclampsia, No. (%) | |  |  | | 0.917 | |  |  | | 0.165 |
| No | | 137 (86.7) | 126 (86.3) | |  | | 118 (81.4) | 61 (73.5) | |  |
| Yes | | 21 (13.3) | 20 (13.7) | |  | | 27 (18.6) | 22 (26.5) | |  |
| Eclampsia with pregnancy/delivery, No. (%) | |  |  | | 0.035 | |  |  | | 0.006 |
| No | | 148 (93.7) | 126 (86.3) | |  | | 134 (92.4) | 66 (79.5) | |  |
| Yes | | 10 (6.3) | 20 (13.7) | |  | | 11 (7.6) | 17 (20.5) | |  |
| Eclampsia with pre-existing HTN, No. (%) | |  |  | | 0.899 | |  |  | | 0.809 |
| No | | 153 (96.8) | 141 (96.6) | |  | | 139 (95.9) | 79 (95.2) | |  |
| Yes | | 5 (3.2) | 5 (3.4) | |  | | 6 (4.1) | 4 (4.8) | |  |
| Postpartum haemorrhage, No. (%) | |  |  | | 0.018 | |  |  | | 0.215 |
| No | | 146 (92.4) | 145 (99.3) | |  | | 129 (89.0) | 78 (94.0) | |  |
| Yes | | 12 (7.6) | 1 (0.7) | |  | | 16 (11.0) | 5 (6.0) | |  |
| Multiple pregnancies, No. (%) | |  |  | | 0.890 | |  |  | | 0.432 |
| No | | 151 (95.6) | 140 (95.9) | |  | | 122 (84.1) | 73 (88.0) | |  |
| Yes | | 7 (4.4) | 6 (4.1) | |  | | 23 (15.9) | 10 (12.0) | |  |
| Premature rupture of membranes, No. (%) | |  |  | | 0.426 | |  |  | | 0.120 |
| No | | 147 (93.0) | 139 (95.2) | |  | | 129 (89.0) | 79 (95.2) | |  |
| Yes | | 11 (7.0) | 7 (4.8) | |  | | 16 (11.0) | 4 (4.8) | |  |
| Type I respiratory failure, No. (%) | |  |  | | 0.006 | |  |  | | 1.000 |
| No | | 155 (98.1) | 131 (89.7) | |  | | 144 (99.3) | 83 (100.0) | |  |
| Yes | | 3 (1.9) | 15 (10.3) | |  | | 1 (0.7) | 0 (0.0) | |  |
| Type II respiratory failure, No. (%) | |  |  | | 0.999 | |  |  | | 1.000 |
| No | | 158 (100.0) | 144 (98.6) | |  | | 144 (99.3) | 83 (100.0) | |  |
| Yes | | 0 (0.0) | 2 (1.4) | |  | | 1 (0.7) | 0 (0.0) | |  |
| Arrhythmia, No. (%) | |  |  | | <0.001 | |  |  | | <0.001 |
| No | | 115 (72.8) | 74 (50.7) | |  | | 111 (76.6) | 43 (51.8) | |  |
| Yes | | 43 (27.2) | 72 (49.3) | |  | | 34 (23.4) | 40 (48.2) | |  |
| Patent ductus arteriosus, No. (%) | |  |  | | 0.862 | |  |  | | 0.037 |
| No | | 152 (96.2) | 141 (96.6) | |  | | 142 (97.9) | 76 (91.6) | |  |
| Yes | | 6 (3.8) | 5 (3.4) | |  | | 3 (2.1) | 7 (8.4) | |  |
| Ventricular septal defect, No. (%) | |  |  | | 0.527 | |  |  | | 0.759 |
| No | | 128 (81.0) | 114 (78.1) | |  | | 131 (90.3) | 76 (91.6) | |  |
| Yes | | 30 (19.0) | 32 (21.9) | |  | | 14 (9.7) | 7 (8.4) | |  |
| Atrial septal defect, No. (%) | |  |  | | 0.480 | |  |  | | 0.390 |
| No | | 110 (69.6) | 107 (73.3) | |  | | 125 (86.2) | 68 (81.9) | |  |
| Yes | | 48 (30.4) | 39 (26.7) | |  | | 20 (13.8) | 15 (18.1) | |  |
| Pulmonary embolism, No. (%) | |  |  | | 0.627 | |  |  | | 1.000 |
| No | | 155 (98.1) | 142 (97.3) | |  | | 144 (99.3) | 83 (100.0) | |  |
| Yes | | 3 (1.9) | 4 (2.7) | |  | | 1 (0.7) | 0 (0.0) | |  |
| Myocardiopathy, No. (%) | |  |  | | 0.346 | |  |  | | 0.911 |
| No | | 152 (96.2) | 137 (93.8) | |  | | 143 (98.6) | 82 (81.9) | |  |
| Yes | | 6 (3.8) | 9 (6.2) | |  | | 2 (1.4) | 1 (1.1) | |  |
| Rheumatic heart disease, No. (%) | |  |  | | 0.984 | |  |  | | 0.367 |
| No | | 146 (92.4) | 135 (92.5) | |  | | 140 (96.6) | 78 (94.0) | |  |
| Yes | | 12 (7.6) | 11 (7.5) | |  | | 5 (3.4) | 5 (6.0) | |  |
| Congenital heart disease, No. (%) | |  |  | | 0.562 | |  |  | | 0.066 |
| No | | 63 (39.9) | 63 (43.2) | |  | | 103 (71.0) | 49 (59.0) | |  |
| Yes | | 95 (60.1) | 83 (56.8) | |  | | 42 (29.0) | 34 (41.0) | |  |
| Eisenmenger syndrome, No. (%) | |  |  | | 0.327 | |  |  | | 0.186 |
| No | | 144 (91.1) | 128 (87.7) | |  | | 138 (95.2) | 82 (98.8) | |  |
| Yes | | 14 (8.9) | 18 (12.3) | |  | | 7 (4.8) | 1 (1.2) | |  |
| Gestational diabetes mellitus, No. (%) | |  |  | | 0.106 | |  |  | | 0.121 |
| No | | 137 (86.7) | 135 (92.5) | |  | | 120 (82.8) | 75 (90.4) | |  |
| Yes | | 21 (13.3) | 11 (7.5) | |  | | 25 (17.2) | 8 (9.6) | |  |
| Infection, No. (%), | |  |  | | 0.011 | |  |  | | 0.029 |
| No | | 148 (93.7) | 123 (84.2) | |  | | 121 (83.4) | 59 (71.1) | |  |
| Yes | | 10 (6.3) | 23 (15.8) | |  | | 24 (16.6) | 24 (28.9) | |  |
| Systemic lupus erythematosus, No. (%) | |  |  | | 0.627 | |  |  | | 0.646 |
| No | | 155 (98.1) | 142 (97.3) | |  | | 141 (97.2) | 79 (95.2) | |  |
| Yes | | 3 (1.9) | 4 (2.7) | |  | | 4 (2.8) | 4 (4.8) | |  |
| Liver insufficiency, No. (%) | |  |  | | 0.999 | |  |  | | 0.007 |
| No | | 154 (97.5) | 146 (100.0) | |  | | 144 (99.3) | 74 (89.2) | |  |
| Yes | | 4 (2.5) | 0 (0.0) | |  | | 1 (0.7) | 9 (10.8) | |  |
| Left to right shunt, No. (%) | |  |  | | 0.827 | |  |  | | 0.088 |
| No | | 153 (96.8) | 142 (97.3) | |  | | 135 (93.1) | 82 (98.8) | |  |
| Yes | | 5 (3.2) | 4 (2.7) | |  | | 10 (6.9) | 1 (1.2) | |  |
| Right-to-left shunt, No. (%) | |  |  | | 0.304 | |  |  | | 0.999 |
| No | | 157 (99.4) | 143 (97.9) | |  | | 142 (97.9) | 83 (81.9) | |  |
| Yes | | 1 (0.6) | 3 (2.1) | |  | | 3 (2.1) | 0 (1.1) | |  |
| Premature delivery, No. (%) | |  |  | | 0.109 | |  |  | | <0.001 |
| No | | 86 (54.4) | 66 (45.2) | |  | | 84 (57.9) | 26 (31.3) | |  |
| Yes | | 72 (45.6) | 80 (54.8) | |  | | 61 (42.1) | 57 (68.7) | |  |
| PH classification, No. (%) | |  |  | | 0.189 | |  |  | | <0.001 |
| Group 1 | | 139 (88.0) | 114 (78.1) | |  | | 139 (95.9) | 60 (72.3) | |  |
| Group 2 | | 16 (10.1) | 24 (16.4) | |  | | 5 (3.4) | 14 (16.9) | |  |
| Group 3 | | 1 (0.6) | 1 (0.7) | |  | | 0 (0.0) | 2 (2.4) | |  |
| Group 4 | | 1 (0.6) | 1 (0.7) | |  | | 0 (0.0) | 0 (0.0) | |  |
| Group 5 | | 1 (1.6) | 6 (4.1) | |  | | 1 (0.7) | 7 (8.4) | |  |
| Pregnancy outcome, No. (%) | |  |  | | <0.001 | |  |  | | 0.016 |
| Vaginal delivery | | 15 (9.5) | 11 (7.5) | |  | | 21 (14.5) | 5 (6.0) | |  |
| spinal and/or epidural for C-section | | 140 (88.6) | 100 (68.5) | |  | | 121 (83.4) | 70 (84.3) | |  |
| general anaesthesia for C-section | | 3 (1.9) | 35 (24.0) | |  | | 3 (2.1) | 8 (9.6) | |  |
| Cardiac surgery, No. (%) | |  |  | | 0.469 | |  |  | | 0.614 |
| No | | 153 (96.8) | 139 (95.2) | |  | | 130 (89.7) | 78 (94.0) | |  |
| Single lung transplantation | | 0 (0.0) | 0 (0.0) | |  | | 5 (3.4) | 0 (0.0) | |  |
| Repair of heart defect | | 5 (3.2) | 7 (4.8) | |  | | 10 (6.9) | 5 (6.0) | |  |
| NYHA functional class, No. (%) | |  |  | | 0.010 | |  |  | | <0.001 |
| I / II | | 109 (69.0) | 75 (51.4) | |  | | 122 (84.1) | 47 (56.6) | |  |
| III | | 30 (19.0) | 44 (30.1) | |  | | 12 (8.3) | 22 (26.5) | |  |
| IV | | 19 (12.0) | 27 (18.5) | |  | | 11 (7.6) | 14 (16.9) | |  |
| NT-proBNP, No. (%), ng/L | |  |  | | 0.001 | |  |  | | 0.190 |
| <1400 | | 133 (84.2) | 99 (67.8) | |  | | 122 (84.1) | 64 (77.1) | |  |
| $\geq$1400 | | 25 (15.8) | 47 (32.2) | |  | | 23 (15.9) | 19 (22.9) | |  |
| Gestation times, median (IQR), times | | 2.0 (1.0-3.0) | 2.0 (1.0-3.0) | | 0.377 | | 2.0 (1.0-3.0) | 2.0 (1.0-3.0) | | 0.555 |
| Parity, median (IQR), times | | 1.0 (1.0-2.0) | 1.0 (1.0-2.0) | | 0.001 | | 2.0 (1.0-2.0) | 1.0 (1.0-2.0) | | 0.746 |
| Troponin, median (IQR), ng/mL | | 0.01 (0.00-0.02) | 0.01 (0.00-0.03) | | 0.267 | | 0.00 (0.00-0.03) | 0.01 (0.01-7.20) | | 0.417 |
| Prothrombin time, median (IQR), s | | 10.2 (9.6-10.8) | 9.8 (9.3-10.6) | | 0.993 | | 10.5 (10.0-11.1) | 10.1 (9.4-11.1) | | 0.046 |
| APTT, median (IQR), s | | 28.7 (26.3-31.2) | 29.2 (26.9-32.3) | | 0.091 | | 27.3 (25.7-30.2) | 28.2 (26.5-31.1) | | 0.035 |
| Thrombin time, median (IQR), s | | 14.2 (12.9-16.3) | 13.6 (12.5-15.5) | | 0.453 | | 14.9 (13.4-16.7) | 13.7 (12.9-14.9) | | 0.327 |
| Fibrinogen, median (IQR), g/L | | 3.8 (3.2-4.5) | 3.6 (3.0-4.0) | | 0.001 | | 3.7 (3.3-4.3) | 3.8 (3.3-4.4) | | 0.947 |
| RBC, median (IQR), * 10^12/L | | 3.9 (3.5-4.3) | 3.9 (3.5-4.2) | | 0.655 | | 3.8 (3.5-4.2) | 3.8 (3.3-4.2) | | 0.715 |
| Haemoglobin, median (IQR), g/L | | 115.0 (102.0-125.0) | 109.0 (100.8-123.0) | | 0.200 | | 109.0 (100.2-118.0) | 112.1 (96.0-123.0) | | 0.253 |
| Platelet, median (IQR), * 10^9/L | | 179.0 (132.0-222.0) | 208.0 (132.8-254.3) | | 0.024 | | 181.0 (144.5-228.5) | 181.0 (126.0-234.0) | | 0.716 |
| D-Dimer, median (IQR), mg/L | | 0.8 (0.4-1.4) | 0.6 (0.3-1.2) | | 0.221 | | 1.0 (0.4-2.6) | 0.6 (0.3-1.0) | | 0.011 |
| RVD, median (IQR), mm | | 22.0 (18.0-29.0) | 21.0 (17.1-26.0) | | 0.050 | | 21.0 (18.0-24.0) | 21.0 (18.0-26.0) | | 0.374 |
| LVDs, median (IQR), mm | | 31.0 (28.0-40.0) | 43.0 (36.0-48.0) | | <0.001 | | 36.0 (30.0-44.9) | 43.0 (40.0-47.0) | | <0.001 |
| mPAD, median (IQR), mm | | 22.0 (21.0-26.0) | 22.0 (20.0-25.0) | | 0.050 | | 23.0 (22.0-26.0) | 23.0 (21.0-26.0) | | 0.274 |
| AOD, median (IQR), mm | | 21.8 (20.0-26.0) | 20.0 (19.0-25.0) | | 0.050 | | 25.0 (21.8-26.0) | 25.0 (20.0-25.0) | | 0.022 |
| EF, median (IQR), % | | 62.0 (59.0-66.0) | 63.0 (60.0-65.0) | | 0.357 | | 64.0 (60.0-68.0) | 63.0 (60.0-65.0) | | 0.096 |
| PASP, median (IQR), mm Hg | | 50.5 (44.0-72.5) | 57.0 (42.0-90.5) | | 0.020 | | 41.0 (36.4-51.0) | 50.0 (38.0-79.0) | | <0.001 |

Two-tailed *P* values < 0.05 were considered statistically significant.

Abbreviations: APTT, activated partial thromboplastin time; AOD, aortic diameter; C-section, Caesarean section; EF, Ejection Fractions; HTN, hypertension; IQR, Interquartile ratio; LVDs, left ventricular systolic diameter; mPAD, mean pulmonary artery diameter; NYHA, New York Heart Association; NT-proBNP, N-terminal pro-brain natriuretic peptide; PASP, pulmonary artery systolic pressure; PH, pulmonary hypertension; RBC, Red blood cells; RVD, right ventricular diameter.

**SUPPLEMENTARY FIGURES**


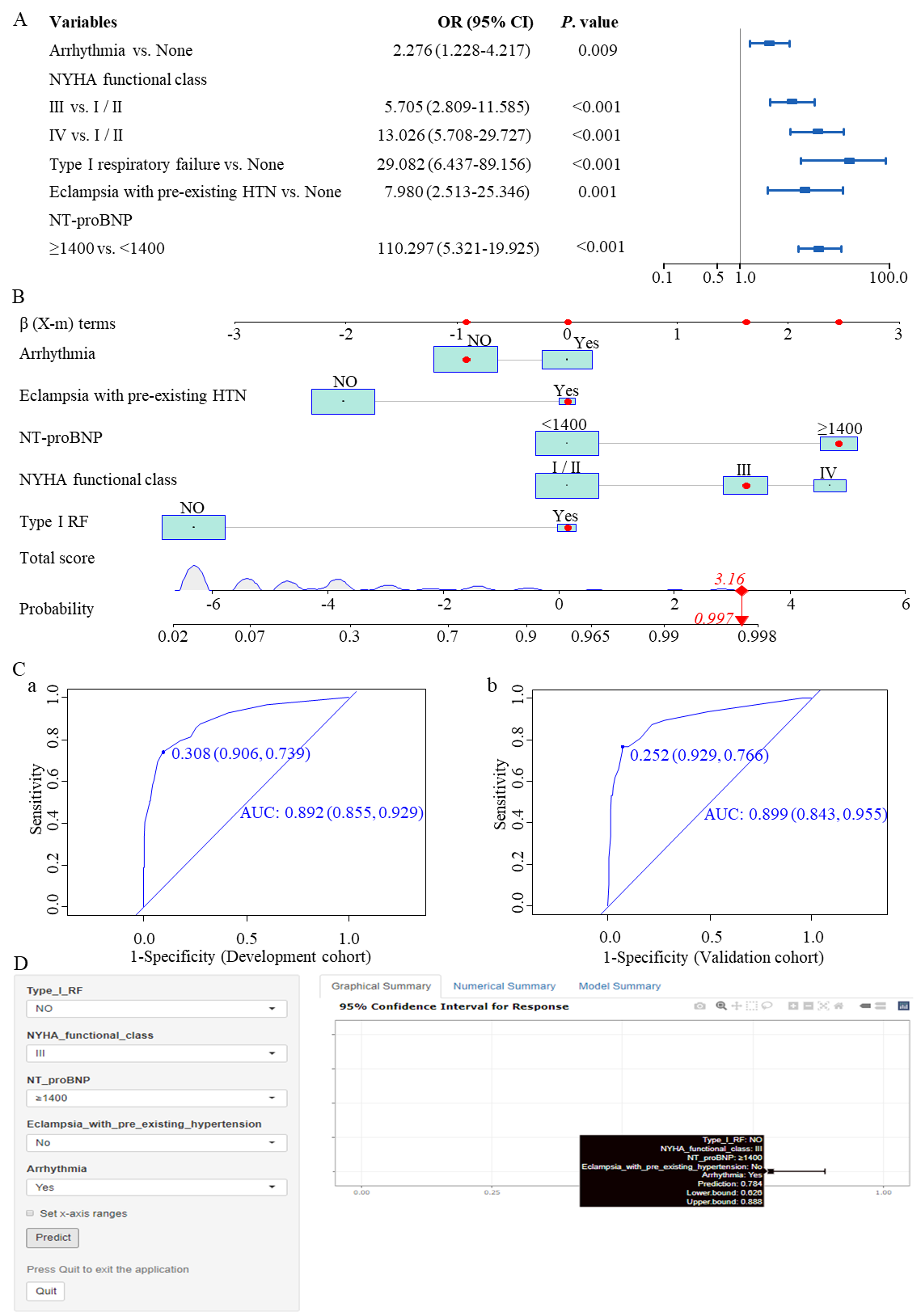


**Fig S1. Forest plot, interactive nomogram, ROC curves, and online dynamic nomogram of Development cohort.**

***A:*** Forest plot showing multivariate logistic regression analysis of the potential risk of maternal mortality or heart failure in pregnant women with PH. ***B:*** Interactive nomogram of the Development cohort. One patient with PH during pregnancy had NYHA function of class III, NT-proBNP level greater than 1400 ng/L, eclampsia with pre-existing hypertension and type I respiratory failure, but no arrhythmia. The predicted maternal mortality or heart failure of this patient in the nomogram is 0.997. ***C (a-b):*** ROC curves of the nomogram in the Development (a) and Validation (b) cohorts, respectively. ***D:*** Online dynamic nomogram is accessible at <https://ph-666.shinyapps.io/maternal-D/>. An example of predicting maternal mortality or heart failure in nomogram is described: The NYHA functional class of the patient was classified as class III, and the level of NT-proBNP was higher than that of 1400 ng/L. Arrhythmia occurred during pregnancy, but she did not have type I respiratory failure and eclampsia with pre-existing hypertension. The Online prediction nomogram predicts a probability of maternal death or heart failure for this patient is 0.784 (95% CI: 0.626–0.888). Abbreviations: CI, confidence interval; HTN, hypertension; NT-proBNP, N-terminal pro-brain natriuretic peptide; NYHA, New York Heart Association; OR, odds ratio; PH, pulmonary hypertension; RF, respiratory failure; ROC, receiver operating characteristic.


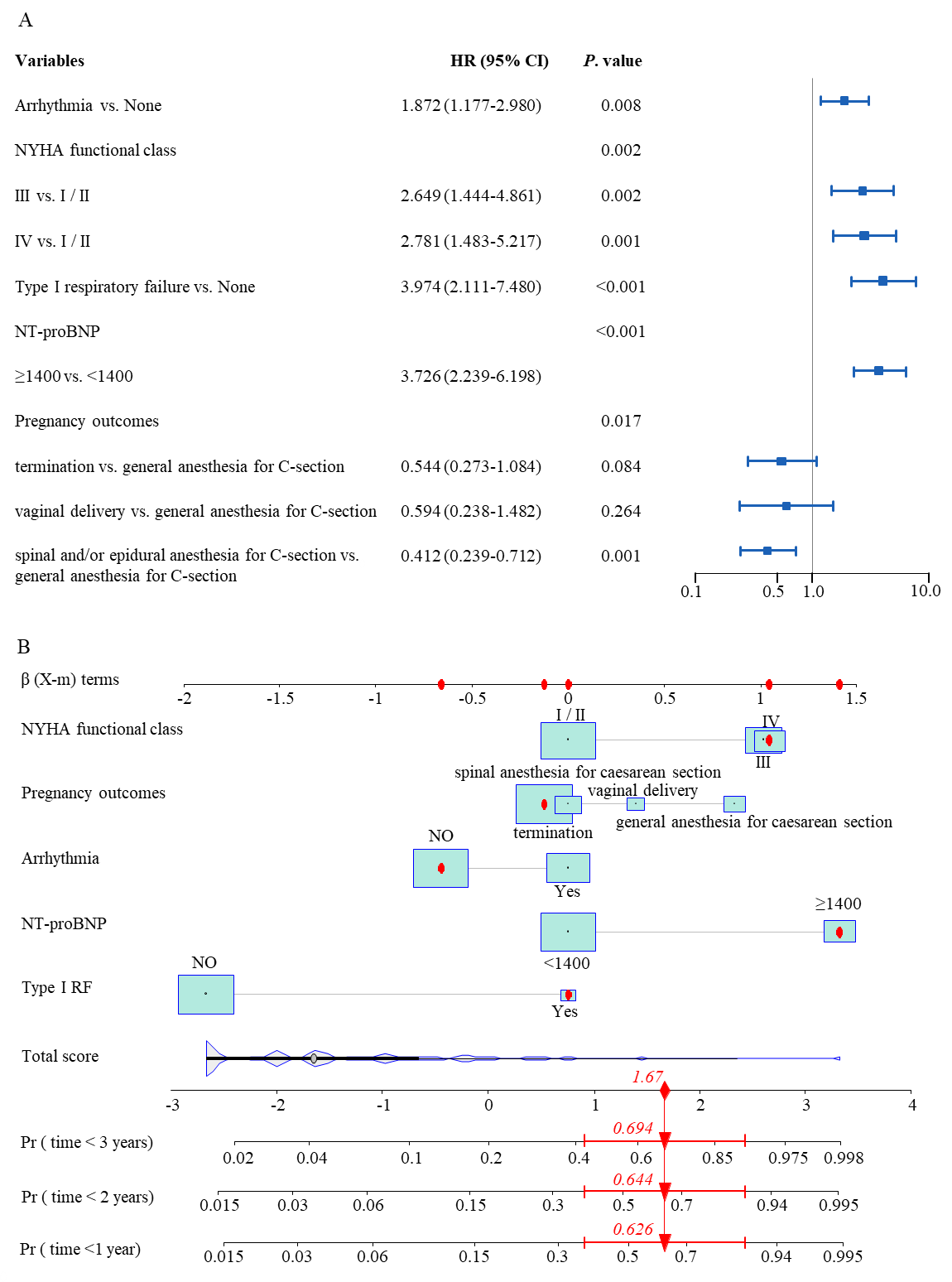


**Fig S2. Forest plot and interactive nomogram of the Follow-up set.**

***A:*** Forest plot showing multivariate cox regression analysis of prognosis risk in pregnant women with PH. ***B:*** Interactive nomogram of the Follow-up set. One patient with PH during pregnancy had NYHA function of class IV and the level of NT-proBNP was higher than that of 1400 ng/L. Type I respiratory failure occurred during pregnancy and spinal anesthesia for caesarean section was performed. No arrhythmias occurred during pregnancy. In this prognostic nomogram, the predicted rates of death and heart failure at 1, 2, and 3 years were 62.6%, 64.4%, and 69.4%, respectively. Abbreviations: CI, confidence interval; C-section, Caesarean section; HR, hazard ratio; NT-proBNP, N-terminal pro-brain natriuretic peptide; NYHA, New York Heart Association; PH, pulmonary hypertension; RF, respiratory failure.


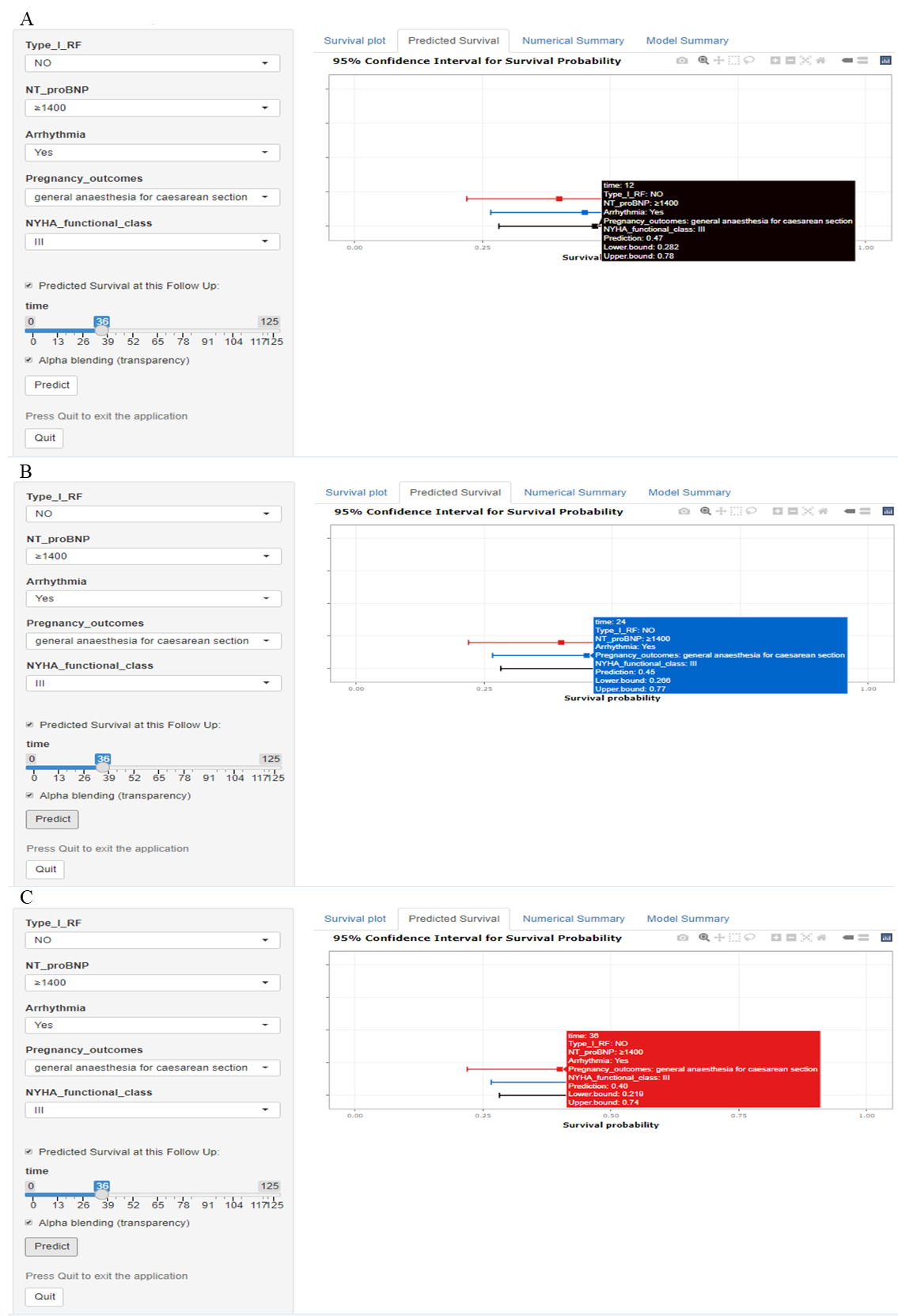


**Fig S3. Online Web software in Follow-up set.**

The online dynamic nomogram is accessible at <https://ph-666.shinyapps.io/COX-pregnant/>. One patient with PH during pregnancy had NYHA function of class III and the level of NT-proBNP was higher than that of 1400 ng/L. There was arrhythmia during pregnancy, but no type I respiratory failure, and general anesthesia for caesarean section was used for delivery. Online prognostic plots showed that the probabilities of 1-year (12 months), 2-year (24 months), and 3-year (36 months) survival and absence of heart failure were 0.47 (95% CI: 0.282–0.78), 0.45 (95% CI: 0.266–0.77), and 0.40 (95% CI: 0.219–0.74), respectively. Abbreviations: CI, confidence interval; NT-proBNP, N-terminal pro-brain natriuretic peptide; NYHA, New York Heart Association; PH, pulmonary hypertension; RF, respiratory failure.


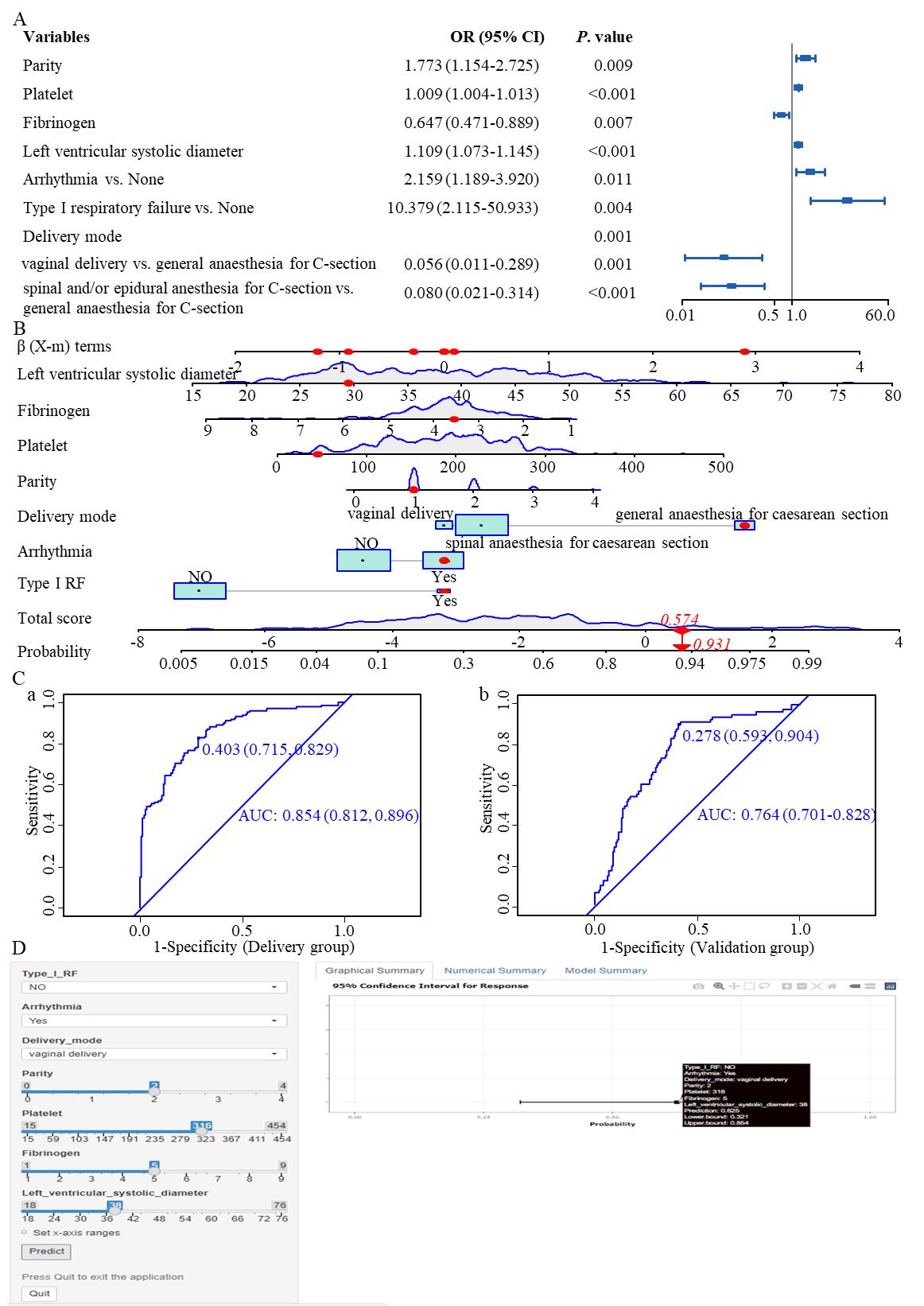


**Fig S4. Forest plot, interactive nomogram, ROC curves, and online dynamic nomogram of Delivery group.**

***A:*** Forest plot showing multivariate logistic regression analysis of the potential risk of adverse foetal/neonatal events in pregnant women with PH. ***B:*** Interactive nomogram of the Delivery group. The fibrinogen of one patient with PH during pregnancy was 3.54 g/L, the platelet count was 46*10^9/L, the left ventricular systolic diameter was 29.49 mm, and the parity was 1. Type I respiratory failure and arrhythmia occurred during pregnancy and were delivered under general anesthesia. The predicted adverse foetal/neonatal event of this patient in the nomogram is 0.931. ***C*** ***(a-b):*** ROC curves of the nomogram in the Delivery (a) and Validation (b) groups, respectively. ***D:*** Online dynamic nomogram accessible at <https://ph-666.shinyapps.io/AE-fetal/>. One patient with PH during pregnancy had fibrinogen 5.0 g/L, platelet count 316*10^9/L, left ventricular systolic diameter 38 mm, and two parities. Arrhythmia but no type I respiratory failure occurred during pregnancy and vaginal delivery were performed. The online prediction nomogram predicts a probability of adverse foetal/neonatal events for this patient is 0.625 (95% CI: 0.321–0.854). Abbreviations: CI, confidence interval; OR, odds ratio; PH, pulmonary hypertension; RF, respiratory failure; ROC, receiver operating characteristic.
